# Functional and Structural Network Recovery after Mild Traumatic Brain Injury: A 1-Year Longitudinal Study

**DOI:** 10.3389/fnhum.2017.00280

**Published:** 2017-05-30

**Authors:** Patrizia Dall’Acqua, Sönke Johannes, Ladislav Mica, Hans-Peter Simmen, Richard Glaab, Javier Fandino, Markus Schwendinger, Christoph Meier, Erika J. Ulbrich, Andreas Müller, Hansruedi Baetschmann, Lutz Jäncke, Jürgen Hänggi

**Affiliations:** ^1^Bellikon Rehabilitation ClinicBellikon, Switzerland; ^2^Division Neuropsychology, Department of Psychology, University of ZurichZurich, Switzerland; ^3^Division of Trauma Surgery, University Hospital ZurichZurich, Switzerland; ^4^Department of Surgery, Division of Traumatology, Kantonsspital AarauAarau, Switzerland; ^5^Department of Neurosurgery, Kantonsspital AarauAarau, Switzerland; ^6^Interdisciplinary Emergency Centre, Baden Cantonal HospitalBaden, Switzerland; ^7^Department of Surgery, Waid Hospital ZurichZurich, Switzerland; ^8^Institute of Diagnostic and Interventional Radiology, University Hospital ZurichZurich, Switzerland; ^9^Brain and Trauma Foundation GrisonChur, Switzerland; ^10^International Normal Aging and Plasticity Imaging Center, University of ZurichZurich, Switzerland; ^11^University Research Priority Program, Dynamic of Healthy Aging, University of ZurichZurich, Switzerland

**Keywords:** mild traumatic brain injury, longitudinal, network recovery, functional connectivity, structural connectivity, graph theoretical analysis, plasticity

## Abstract

Brain connectivity after mild traumatic brain injury (mTBI) has not been investigated longitudinally with respect to both functional and structural networks together within the same patients, crucial to capture the multifaceted neuropathology of the injury and to comprehensively monitor the course of recovery and compensatory reorganizations at macro-level. We performed a prospective study with 49 mTBI patients at an average of 5 days and 1 year post-injury and 49 healthy controls. Neuropsychological assessments as well as resting-state functional and diffusion-weighted magnetic resonance imaging were obtained. Functional and structural connectome analyses were performed using network-based statistics. They included a cross-sectional group comparison and a longitudinal analysis with the factors group and time. The latter tracked the subnetworks altered at the early phase and, in addition, included a whole-brain group × time interaction analysis. Finally, we explored associations between the evolution of connectivity and changes in cognitive performance. The early phase of mTBI was characterized by a functional hypoconnectivity in a subnetwork with a large overlap of regions involved within the classical default mode network. In addition, structural hyperconnectivity in a subnetwork including central hub areas such as the cingulate cortex was found. The impaired functional and structural subnetworks were strongly correlated and revealed a large anatomical overlap. One year after trauma and compared to healthy controls we observed a partial normalization of both subnetworks along with a considerable compensation of functional and structural connectivity subsequent to the acute phase. Connectivity changes over time were correlated with improvements in working memory, divided attention, and verbal recall. Neuroplasticity-induced recovery or compensatory processes following mTBI differ between brain regions with respect to their time course and are not fully completed 1 year after trauma.

## Introduction

Mild traumatic brain injury (mTBI) has an annual incidence of 100–300 cases per 100,000 persons, if counting only patients treated in hospitals (Cassidy et al., 2004). Although the majority of patients have a spontaneous history of favorable remission, mTBI can lead to long-term symptoms characterized by cognitive, emotional, and physical disturbances. These symptoms are referred to as post-concussion disorder (PCD). It is increasingly being recognized that conventional diagnostic neuroimaging and measures of cognitive function are not suitable to predict outcomes and neuronal compensation after mTBI. Graph theoretical analysis supplies a straightforward way to evaluate complex neuronal networks (Bullmore and Sporns, 2009; Zalesky et al., 2010) as well as changes in connectivity following disrupted neuronal systems (Nakamura et al., 2009). In this respect, neuroimaging studies that combine functional and structural investigations of neuronal networks provide a more comprehensive picture of the neuroplasticity after mTBI. Furthermore, there have been only a few multimodal imaging studies aimed at elucidating the transitions between early and later stages of mTBI compared to the course of the healthy brain. Longitudinal magnetic resonance imaging (MRI) studies focusing on connectivity changes after mTBI have already been described, but these reported mixed findings, investigated only one MRI modality and had short follow-up periods. For example, some restoration of network dysfunction has been described over a period of 6 months after mTBI using longitudinal resting state functional MRI (rsfMRI) within a specific frequency range in the default mode network (DMN; Sours et al., 2015a). During the same time window, another study revealed recovery from diffuse decreased connectivity in the acute phase after injury, with the majority of the changes seen between 3 and 6 months and not between 0 and 3 months (Bharath et al., 2015). A trend of slow normalization within the DMN was even reported in a small pilot study with concussed athletes from day 7 to day 30 compared with the control group (Zhu et al., 2015). Mainly due to the very limited number of longitudinal connectomic studies, the relationship between restored functional connectivity over time and the corresponding changes in measures of cognitive functioning is just beginning to be explored (Bharath et al., 2015). On the contrary, other longitudinal studies did not reveal any functional recovery of initially decreased connectivity within the DMN and greater connectivity between the DMN and the prefrontal cortex (PFC) across a 4-month (Mayer et al., 2011) or a 6-month recovery period (Sours et al., 2015b).

In the existing mTBI literature, we did not identify studies looking at the longitudinal reorganization of structural network topology. Complementary, classical diffusion tensor imaging (DTI) studies reported about equal evidence of both increased and decreased fractional anisotropy (FA) in adult samples during the semi-acute phase (Dodd et al., 2014). However, when focusing on studies conducted in acute mTBI rather increased FA has been revealed, while decreased FA findings are reported more frequently for post-acute studies (Eierud et al., 2014).

Studies of functional and structural connectivity changes during recovery after moderate and severe traumatic brain injury (TBI) are also relatively sparse and recent. By examining regional changes in the DMN, functional alteration has been demonstrated during a recovery period of 6 months after severe TBI (Hillary et al., 2011). The patients improved their performance on a working memory task and showed increased connectivity in the posterior cingulate cortex (PCC) and medial PFC, regions associated with internal-state responsivity. Another study on severe TBI aimed at delineating the progression of traumatic axonal injury in major fiber tracts over 6–11 months post-injury found that tractography-based measurements, which improved, remained stable, or deteriorated further, correlated with patients’ long-term outcome ranging from good recovery to severely disabled (Wang et al., 2011). Nevertheless, all investigated white matter tracts showed systematic deterioration at group level.

We conducted a 1-year prospective study that tracks large-scale functional and structural network alterations in a cohort of mTBI patients compared to healthy controls. In addition, we explored how alterations of functional and structural connectivity are related to each other and how they are related to changes in cognition. A final objective of the current study was to determine whether mTBI patients with PCD would exhibit a different recovery trajectory in functional and structural connectivity, when compared with those without PCD.

## Materials and Methods

### Participants: Demographic and Clinical Data

A total of 60 patients with mTBI and 58 healthy controls matched for gender, age, and education were prospectively recruited between February 2012 and March 2014. Healthy subjects were recruited through public advertising and among acquaintances of researchers and staff. The diagnosis of mTBI was given in accordance with the European Federation of Neurological Societies guidelines (Vos et al., 2012) using standardized criteria across the emergency departments of four hospitals in the German region of Switzerland. Inclusion criteria comprised (1) a Glasgow Coma Scale (GCS) score of 13–15 at hospital admission; (2) a normal cranial computed tomography (CT); (3) at least one of the following characteristics: loss of consciousness <30 min; presence of a qualitative alteration in mental status such as confusion, disorientation, or dizziness at the time of incident; post-traumatic amnesia <60 min; and retrograde amnesia <30 min; and (4) age ranging between 18 and 64 years. Exclusion criteria were as follows: history of neurologic or psychiatric disease, neurosurgery, previous TBI, attention-deficit/hyperactivity disorder (ADHD), current or previous drug or alcohol abuse, and contraindications to MRI. A previous mTBI in the preceding 3 months before investigation was an exclusion criteria. Nine patients and five controls were excluded because of incidental brain anomalies (two patients and one control), excessive MRI-related motion artifacts (two patients), history of prior neurologic or psychiatric disturbance (two patients and three controls), questionable diagnosis of mTBI (one patient) and uncertain follow-up participation (two patients and one control). This resulted in a sample of 51 patients and 53 controls. Patients were investigated clinically and brain images were acquired within 7 days post-injury (Visit 1, acute phase) and reinvestigated 1 year later (Visit 2, referred to as chronic phase). Within the same time interval, control subjects completed the identical assessments as the patients, expect for the neurological examination carried out by neurologists. Only participants who completed the entire investigation process across the two visits and were free of structural anomalies on conventional MR images were admitted for the longitudinal analysis reported here. Of the initial sample, 49 patients and all 53 controls returned for the follow-up visit (one patient moved abroad and another one was pregnant at Visit 2). To guarantee an exact one-to-one matching of patients and controls, the size of the control cohort was further restricted to 49 subjects. The four artificially excluded healthy subjects were representative of our healthy control group. The average time interval between scans was 365.9 days (SD = 4.0 days) for the patients as well as 364.6 days (SD = 5.1 days) for the controls. All subjects were equally reimbursed to make up for income lost due to study participation. Two Swiss Cantonal Ethics Committees (Ethics Committee Zurich and Ethics Committee for Northwest/Central Switzerland) approved the study protocol and all participants provided written informed consent prior to their participation.

### Neuropsychological Assessment

A full battery of standardized and validated neuropsychological tests designed to be sensitive to diverse cognitive impairments commonly observed following mTBI was applied. These tests included measures of attention, executive functions, working memory, verbal memory as well as intelligence and measures of effort. In addition, clinical questionnaires were used to assess mTBI-related symptomatology as well as to control reactive manifestations of depression and anxiety. The descriptions of cognitive tests and clinical questionnaires are provided in the Supplementary Material. A PCD was established at Visit 2 based on subjective symptoms from the Rivermead Post-Concussion Symptoms Questionnaire (RPQ) (King et al., 1995). The RPQ assesses the most common PCD symptoms spanning cognitive, emotional and physical domains. At Visit 2, patients reporting three or more post-concussion symptoms rated as moderate to severe problems were pooled into a patient subcohort experiencing chronic PCD.

### Image Acquisition Protocols and Preprocessing

Recordings included the following sequences: (a) resting-state T2^∗^-weighted fMRI, (b) volumetric 3D T1-weighted MRI, (c) diffusion-weighted spin echo-planar imaging, (d) T1-weighted B0 map, (e) susceptibility-weighted imaging, (f) dual spin-echo (T2- and proton-density-weighted) as well as (g) fluid attenuated inversion recovery scans. Standard clinical MRI scans were evaluated at both time points by the same certified radiologist in order to detect intraparenchymal pathology (e.g., hemorrhages, tumors) considered as exclusion criteria. Details of functional and structural MRI data acquisition parameters are completely reported in the Supplementary Material.

Preprocessing of rsfMRI and DTI data as well as the construction of functional and structural connectivity networks are described in detail in the Supplementary Material.

### Statistical Analyses

#### Functional and Structural Connectivity Analyses

Connectivity analyses were based on a 90-node whole-brain network constructed from the 90-region automated anatomical labeling atlas (Tzourio-Mazoyer et al., 2002), one node for each region of interest (ROI). Concerning functional connectivity, for each region a mean resting state time series was calculated as the mean over all voxel-time series of the respective region (mean ROI-time series). Functional connectivity measures are partial correlations accounting for white matter, cerebrospinal fluid, global signal, and for 24 head motion parameters (Friston-24). Fisher’s *z*-transformed Pearson’s correlation coefficients of mean ROI-time series pairs were taken as connectivity measure. For structural connectivity analysis, the numbers of streamlines connecting each pair of ROIs were used as connectivity measures. These values in form of connectivity matrices were subject to network-based statistic (NBS). NBS is a validated non-parametric method and a tool for identifying statistically significant subnetworks of a given network (connectivity matrix; Zalesky et al., 2010). Using a family-wise error rate control with *p* < 0.05, NBS accounts simultaneously for multiple hypotheses testing over all edges of a network. To explore the potential linkage between number of streamlines and more traditional diffusion measures, mean FA (averaged across all streamlines connecting two ROIs) of the subnetwork of interest was also calculated. Group comparisons at Visit 1 and interaction analyses were computed directly with NBS with the component extent option, significance level alpha = 0.05 corrected for multiple comparisons, one-sided hypothesis testing and 5,000 permutations per statistical test. The reported *t*-thresholds are sensitivity (set) thresholds (Zalesky et al., 2010) and were used to determine which edges of the connectivity matrix form the largest subnetwork subjected to permutation statistics. These thresholds have to be determined by exploration and are chosen in an arbitrary way. Specifically, the set *t*-threshold does not affect the false positive rate of the actual permutation statistic of the alpha error probability. Results of the network analyses were visualized using the BrainNet Viewer software (Xia et al., 2013). In NBS, we firstly ran a two-sample *t*-test for each of the possible 4,005 (90 × 89/2) connections to capture significant whole-brain difference in functional and structural connectivity between the patient and control groups at Visit 1. We then analyzed group × time interaction using NBS selecting exclusively the subnetwork identified as significant from the differences observed between groups at Visit 1 (selective interaction). For this purpose, we subtracted the Fisher’s *r*-to-*z*-transformed Pearson’s correlation coefficients (functional connectivity) and the number of reconstructed streamlines (structural connectivity) at Visit 2 from those at Visit 1 each resulting in a difference map operationalizing the change over time. If the interaction analyses resulted in significant subnetworks, we exported the data to IBM SPSS statistics software (22.0) and ran 2 (group) × 2 (time) repeated measures analysis of variances (ANOVAs) on connectivity values averaged across all edges of the significant subnetwork. In this way, we uncovered the direction of the interaction within the identified subnetworks as well as the main effects (between and within groups). The selective approach chosen here has the advantage of tracking the impaired subnetwork found at Visit 1, but has the disadvantage of overlooking possible differences not present within the first few days after mTBI. Therefore, we also assessed whole-brain group × time interactions for both functional and structural connectivity, independently from the initial level of network impairment. Finally, functional group differences (mean functional connectivity strength over a significant functional subnetwork) were correlated with structural group differences (mean structural connectivity strength over a significant structural subnetwork) at Visit 1 using Pearson’s correlation. Additional correlations were calculated between functional and structural changes resulting from the selective group × time interactions. These *post hoc* analyses were performed using SPSS and a significance level of 0.05 (two-sided, unless otherwise indicated) was applied. The effect sizes for group comparisons were computed according Cohen’s *d* along with their associated 95% confidence intervals (CIs) and the effect sizes for group × time interactions were computed using partial eta-square and then converted to Cohen’s *d* using formula reported elsewhere (Cohen, 1988). Cohen suggested defining the effect size as small (*d* = 0.2), medium (*d* = 0.5), and large (*d* = 0.8). Within- and between-group comparisons of the head motion parameters have been performed with *t*-tests using SPSS.

#### Associations between Cognitive Performance and Network Metrics

Cross-sectional differences in demographic and neuropsychological characteristics between groups were determined using unpaired *t*-tests and longitudinal differences within each group were performed using paired *t*-tests. Correlations between longitudinal changes in functional and structural mean connectivity strengths and longitudinal changes in cognitive performance were examined within the patients group only. These correlations were conducted using partial correlations controlling for age, education, and total gray matter volume (for functional connectivity) or age, education, and total white matter volume (for structural connectivity). All associations between cognitive performance and network metrics were run in SPSS with partial Spearman rank-order correlations (rho) that also account for the influence of outliers in the neuropsychological measurements.

## Results

### Participant Characteristics and Neuropsychological Measures

**Table 1** summarizes the characteristics of the patients (*n* = 49) and of the healthy controls (*n* = 49) included in the final sample. Note that there were no significant differences in potential demographic confounders or in the time interval of MRI acquisitions between the two groups.

**Table 1:** Characteristics of patient and control groups.

	Visit 1 (acute phase)			Visit 2 (chronic phase)			Repeated measures	
	Patients (*n* = 49), mean (SD)	Controls (*n* = 49), mean (SD)	*p*-Value (patients vs. controls)	Patients (*n* = 49), mean (SD)	Controls (*n* = 49), mean (SD)	*p*-Value (patients vs. controls)	*p*-Value (patients)	*p*-value (controls)
**Demographics and clinical measures**								
Age (years)	34.9 (12.4)	35 (12.1)	0.96	35.9 (12.4)	36 (12.1)	0.96	n/a	n/a
Gender (male/female)	18/31	18/31	1.00	18/31	18/31	1.00	n/a	n/a
Education (years)	12.6 (2.5)	12.9 (2.4)	0.49	12.7 (2.5)	13 (2.5)	0.44	0.02	0.01
Days between scans	–	–	–	365.9 (4.0)	364.6 (5.1)	0.18	–	–
Glasgow Coma Scale	14.8 (0.4)	–	–	–	–	–	–	–
mTBI in the past	0.6 (0.9)	0.4 (0.8)	0.18	–	–	–	–	–
**Global brain measures**								
Total gray matter volume (cm^3^)	650.4 (67.3)	649.1 (55.3)	0.92	649.5 (66.5)	646.7 (54.4)	0.82	0.71	0.15
Total white matter volume (cm^3^)	474.1 (58.8)	478.3 (38.4)	0.68	474.8 (59.6)	477.7 (37.7)	0.77	0.43	0.39
**Global connectivity measures (90 nodes)**								
Number of streamlines	2,159,848	2,147,650	0.78	2,158,364	2,162,031	0.93	0.89	0.08
Streamlines omitted	1,375,985	1,353,384	0.39	1,368,688	1,362,667	0.81	0.41	0.17
Streamlines used to populate matrix	832,699	840,888	0.75	837,946	846,829	0.73	0.55	0.39
Selfloops	468,174	464,983	0.79	468,230	466,147	0.86	0.99	0.65
**Neuropsychological assessment**								
RPQ (total score)	14.2 (10.8)	2.8 (3.9)	<0.001	7 (9.9)	2.5 (4.4)	0.005	<0.001	0.54
Alertness, tonic (ms)	243.2 (53.4)	220.4 (20.5)	0.007	219.2 (20.9)	216.9 (20.2)	0.59	0.004	0.16
Alertness, phasic (ms)	244.7 (70.2)	221.4 (22.8)	0.03	215.9 (18.1)	216 (21.2)	0.99	0.01	0.10
Go/No-go (ms)	398.3 (69.1)	370.1 (45.2)	0.02	380.4 (62.2)	359.2 (46.1)	0.06	0.02	0.04
Go/No-go (errors)	0.9 (1.2)	1.0 (1.1)	0.61	0.9 (0.9)	0.9 (0.9)	0.83	0.73	0.45
Divided attention, auditory (ms)	584.9 (94.4)	559.6 (75.4)	0.15	553.8 (70.9)	532.6 (68.2)	0.13	0.03	0.01
Divided attention, visual (ms)	805.9 (105.1)	756.9 (89.2)	0.01	741.7 (90.7)	706.2 (82.5)	0.05	<0.001	<0.001
Working memory	5.4 (1.2)	5.8 (1.3)	0.05	5.9 (1.4)	6 (1.2)	0.64	0.001	0.39
AVLGT immediate recall score	57.5 (6.9)	59.4 (6.9)	0.18	61.9 (6.1)	63.3 (6.8)	0.33	<0.001	<0.001
AVLGT long delayed	12.9 (1.8)	13.2 (1.9)	0.48	13.9 (1.6)	14.1 (1.2)	0.48	0.002	<0.001
BDI-II (score)	6.4 (5.8)	3.5 (4.4)	0.01	4.1 (4.8)	2.8 (4.3)	0.17	0.003	0.28
BAI (score)	2.8 (6.6)	0.6 (2.2)	0.03	1.9 (4.5)	0.14 (1.0)	0.01	0.40	0.04
Intellectual ability (IQ)	101.2 (15.1)	107.8 (14.5)	0.03	–	–	–	–	–
Performance validity (MSVT)	–	–	–	Good effort	Good effort			

*n/a, not applicable; RPQ, Rivermead Post-Concussion Symptoms Questionnaire; AVLGT, German adaptation of the Rey Auditory Verbal Learning Tests (RAVLT); BDI-II, Beck Depression Inventory, 2nd edition; BAI, Beck Anxiety Inventory; MSVT, Medical Symptom Validity Test.*

The mean age of the patients was 34.9 years (range 18–61 years) and that of the controls was 35 years (range 18–60 years). At arrival in the emergency department, the majority of the patients (*n* = 40) had an initial GCS of 15, eight patients were admitted with 14 and one patient with 13. Neuroimaging and neuropsychological investigations were performed at an average of 4.9 days (SD = 1.5 days) and 5.3 days (SD = 1.6 days) post-injury, respectively. Compared to the well-matched control group, patients with mTBI showed initial worse performance across a subset of cognitive tasks and greater impairments on questionnaire-based clinical measures. In particular, patients exhibited significantly higher symptom severity on the RPQ. At follow-up, cognitive recoveries as well as clinical improvements have been observed, although higher scores of post-concussive symptoms were still measured in patients. Six of 49 patients were identified as having a chronic PCD (about 12%) on the basis of their symptoms reported 1 year after the injury and the remaining 43 patients had a good outcome. We did not observe any differences in age (*p* = 0.19), sex (*p* = 0.65), and GCS (*p* = 0.83) between the patients with and without chronic PCD. However, there was a statistical trend in the years of education (mean/SD: 12.84/2.5 years for good outcome, 10.83/1.5 years for bad outcome, *p* = 0.06) with chronic PCD-patients having fewer years of schooling. All participants showed good effort on tests of symptom validity. The corrected *p*-values for all 49 tests adjusted using false discovery rate (FDR) correction are reported in Supplementary Table 1.

There were no statistically significant group differences in head motion parameters of both the functional and structural MRI scans neither between groups at both time points nor between time points within groups. For the rsfMRI data, mean frame-wise displacements computed according the method proposed by Power et al. (2012, 2015) and for the DTI data, mean frame-wise translations and rotations computed according the method proposed by Yendiki et al. (2014) were small and comparable between groups at both time points and between time-points within groups.

### Functional and Structural Connectivity Analyses—Group Comparison

**FIGURE 1:**
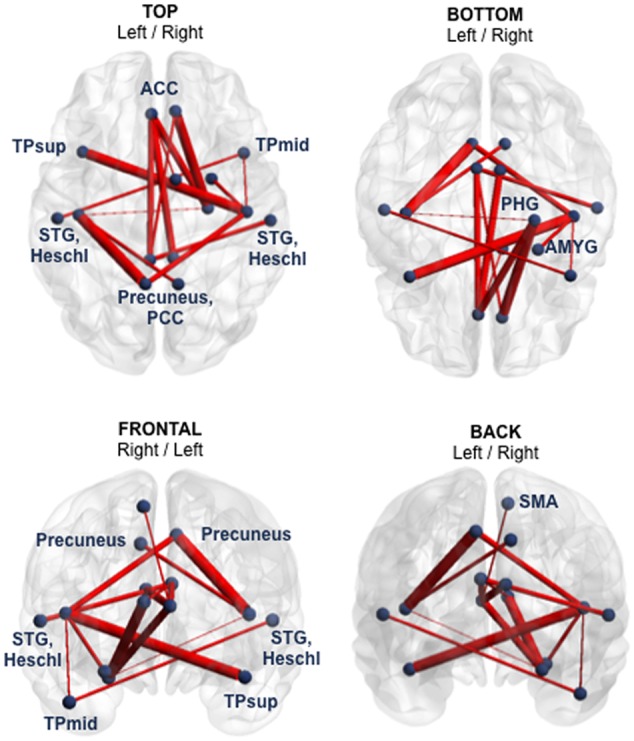
Subnetwork with reduced functional connectivity in the mTBI sample at Visit 1 (group comparison). Blue points correspond to the 15 nodes of the subnetwork and red lines represent the 15 suprathreshold connections that showed reduced functional connectivity in mTBI patients, compared with healthy controls. Cohen’s *d* = 1.59, 95% CI = 1.138–2.046, *p* = 0.0057, corrected for multiple comparisons. The NBS-specific set threshold forming the component was set to *t* = 3.16. More details about the connections of this subnetwork can be found in Supplementary Table 2. ACC, anterior cingulate cortex; AMYG, amygdala; PCC, posterior cingulate cortex; PHG, parahippocampal gyrus; SMA, supplementary motor area; STG, superior temporal gyrus; TPmid, middle temporal pole; TPsup, superior temporal pole.

At Visit 1, functional hypoconnectivity within a 15-edge subnetwork distributed over 15 nodes was detected in the mTBI group compared to controls (Cohen’s *d* = 1.59, 95% CI = 1.138–2.046, *p* = 0.0057). The pattern of this subnetwork with reduced functional connectivity consisted almost exclusively of bilateral structures (12 of 15 nodes) such as the anterior cingulate cortex (ACC), PCC, precuneus, Heschl’s gyrus, superior temporal gyrus (STG) and temporal pole (TP). In addition, the parahippocampal gyrus, amygdala, and supplementary motor area (SMA) were only present in the right hemisphere (**Figure 1** and Supplementary Table 2).

The profile of the altered edges showed 10 inter- and five intra-hemispheric connections. Of note, seven of the 15 edges involved the nodes ACC or PCC. Briefly, the impaired functional subnetwork revealed many key nodes of the classical DMN.

**FIGURE 2:**
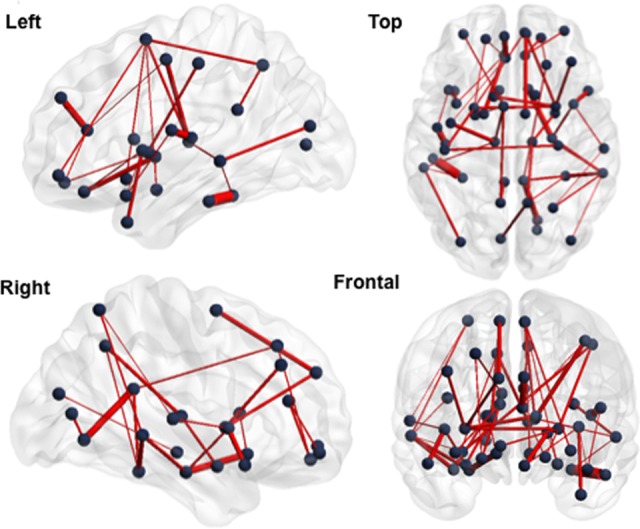
Subnetwork with increased structural connectivity in the mTBI sample at Visit 1 (group comparison). Blue points correspond to the 52 nodes of the subnetwork and red lines represent the 53 suprathreshold connections that showed increased structural connectivity in mTBI patients, compared with healthy controls. Cohen’s *d* = –1.71, 95% CI = –2.168 to –1.243, *p* = 0.041, corrected for multiple comparisons. The NBS-specific set threshold was set to *t* = 1.87. More details about the connections of this subnetwork can be found in Supplementary Table 3.

In the cross-sectional structural analysis, patients showed a widespread increase in connectivity compared to controls (Cohen’s *d* = -1.71, 95% CI = -2.168 to -1.243, *p* = 0.041). Structural hyperconnectivity was detected in a 53-edge subnetwork distributed over 52 nodes comprising connections encompassing frontal, temporal, parietal, occipital, and subcortical regions (**Figure 2** and Supplementary Table 3).

Of the 53 edges, 48 were intra-hemispheric, whereas 36 of the 52 nodes were bilaterally represented, including ACC, PCC, precuneus, and TP. This increase in the number of streamlines between these nodes in the mTBI group also corresponded to a significant increase in FA as well as to a decrease in global efficiency and an increase in normalized characteristic path length compared with healthy controls (see Supplementary Results). No brain area showed functional hyperconnectivity or structural hypoconnectivity in patients relative to controls. We also estimated the reproducibility of the structural finding, which were obtained using the “eddy_correct” tool, with that obtained by employing the “eddy” tool for the correction of eddy currents including parallel the average of the mean of translational and rotational motion estimation parameters as a nuisance regressor in the analysis with NBS. A common artifact in diffusion imaging is signal attenuation caused by macroscopic head motion (Yendiki et al., 2014). We demonstrated reproducible mTBI-related group differences across the different corrections of eddy current-induced distortions (see Supplementary Results). The fact that both results were qualitatively comparable may be attributable to the low *b*-value of 1,000 s/mm^2^ (Andersson and Sotiropoulos, 2016).

### Functional and Structural Connectivity Changes Over Time—Interaction

**FIGURE 3:**
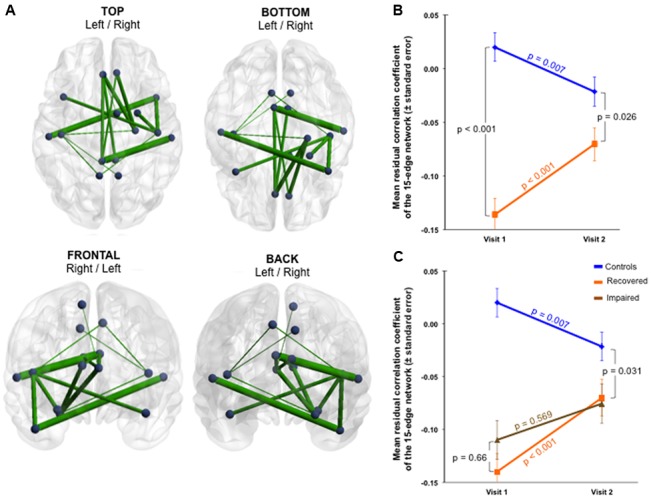
Functional changes over 1 year within the initially impaired 15-edge subnetwork (selective group × time interaction). **(A)** The NBS-specific set threshold was set to *t* = 0 in order to admit all possible connections of the 15-edge subnetwork to the set of suprathreshold links showing a change over time (Cohen’s *d* = 0.9, 95% CI = 0.490–1.321, *p* = 0.002, corrected for multiple comparisons). **(B)** The significant repeated-measures effect resulted from an increase in the mean correlation coefficient within the patient sample along with a decrease within the control sample. The time effect for each group revealed significant evolutions over 1 year. **(C)** The line graph is color-coded orange and brown corresponding to patients with good (*n* = 43) and poor outcome (*n* = 6), respectively. The group difference was smaller at 1 year post-injury when only the 43 patients with good outcome were considered. Repeated measure over the subset of patients with good outcome versus controls revealed a more rapid trajectory toward normalization. At a descriptive level, the recovery curves in patients developing chronic post-concussive syndrome were somewhat flat. More details about the connections of this subnetwork can be found in Supplementary Table 4.

The group × time interaction of the impaired subnetworks defined at Visit 1 (selective interaction) displayed significant results in both functional and structural connectivity. Firstly, a significant functional change was identified in the whole 15-edge subnetwork (Cohen’s *d* = 0.9, 95% CI = 0.490–1.321, *p* = 0.002; **Figure 3A** and Supplementary Table 4).

The exploration of this longitudinally altered subnetwork showed that an increase in the mean functional connectivity within the patient sample and a decrease within the control sample were responsible for the significant interaction observed (**Figure 3B**). The group effect within the 15-edge subnetwork reached a marked difference at Visit 1 that shifted into a weaker, but still statistically significant difference at Visit 2. When only the 43 patients with good outcome were considered, the group difference at 1 year post-injury was smaller (**Figure 3C**). Furthermore, the effect of time for each group separately showed significant evolution in the connections’ strength. The recovery curve in patients with persistent PCD (*n* = 6) was rather flat compared with that of patients with good outcome (*n* = 43), even though statistical analysis on a small sample size should be interpreted with caution.

**FIGURE 4:**
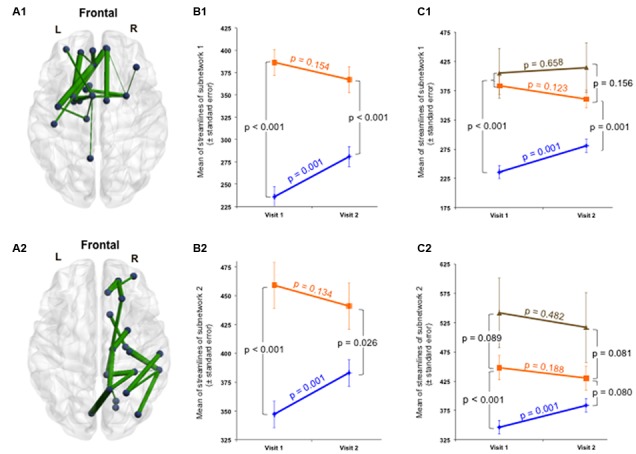
Structural changes over 1 year within the initially impaired 53-edge subnetwork (selective group × time interaction). The NBS-specific set threshold was set to *t* = 0 in order to admit all possible connections of the 53-edge subnetwork to the set of suprathreshold links showing a change over time. **(A1)** Subnetwork 1 encompassed 19 edges and is accentuated toward the left hemisphere (Cohen’s *d* = –0.72, 95% CI = –1.132 to –0.315, *p* = 0.025, corrected for multiple comparisons). **(A2)** Subnetwork 2 comprised 18 edges and is accentuated toward the right hemisphere (Cohen’s *d* = –0.71, 95% CI = –1.120 to –0.303, *p* = 0.035, corrected). **(B1,B2)** A decrease in the number of streamlines in the patient sample and an increase in the control sample were responsible for the significant repeated-measures effect. The time effect was significant in the controls, but only weakly trend in the patients. **(C1,C2)** When focusing on the group analyses of the subcohort of patients without post-concussive syndrome (*n* = 43, orange trajectory) at follow-up, the difference to the control group was no longer significant for the 18-edge subnetwork, but still evident for the 19-edge subnetwork. There was no significant difference between patients with and without PCD at Visit 2. More details about the connections of this subnetwork can be found in Supplementary Table 5.

Secondly, the group × time interaction of structural connectivity within the 53-edge subnetwork resulting from the group comparison at Visit 1 (selective interaction) revealed significant alterations in two distinct subnetworks (Supplementary Table 5). One subnetwork was concentrated in the frontal part of the left hemisphere and showed a profile of 19 edges distributed over 19 nodes (Cohen’s *d* = -0.72, 95% CI = -1.132 to -0.315, *p* = 0.025, subnetwork 1A in **Figure 4**).

The second subnetwork was concentrated in the right hemisphere and consisted of 18 connections distributed over 19 nodes (Cohen’s *d* = -0.71, 95% CI = -1.12 to -0.303, *p* = 0.035, subnetwork 2A in **Figure 4**). The splitting into two subnetworks was due to the fact that only five of 53 connections composing the initially altered subnetwork were inter-hemispheric. Both structural subnetworks demonstrating changes over time revealed decreased connectivity for the patients along with increased connectivity for the controls (**Figure 4**).

At the initial visit, the group effect was strong for both subnetworks. One year later, both structural subnetworks normalized in individuals with mTBI, but the recovery was not complete since the mean of streamlines was still increased compared to controls. No significant difference was found between patients with and without PCD at Visit 2. When focusing on the group analyses on the subcohort of patients without PCD at follow-up, the difference to the control group was no more significant for the 18-edge subnetwork, but still evident for the 19-edge subnetwork. The time effect in the patients with good outcome approached levels of a weak trend, whereas the trajectories of patients with persistent PCD were rather flat for both subnetworks (**Figure 4**). Please note, findings should be interpreted with caution due to the small number of patients with chronic PCD (*n* = 6).

Other figures showing the functional and structural connectivity changes over time at individual level can be found in the Supplementary Figures 5–7.

The group × time interaction analysis of FA indicated a trend toward decreased mean FA for the patients over time and is reported in the Supplementary Results.

**FIGURE 5:**
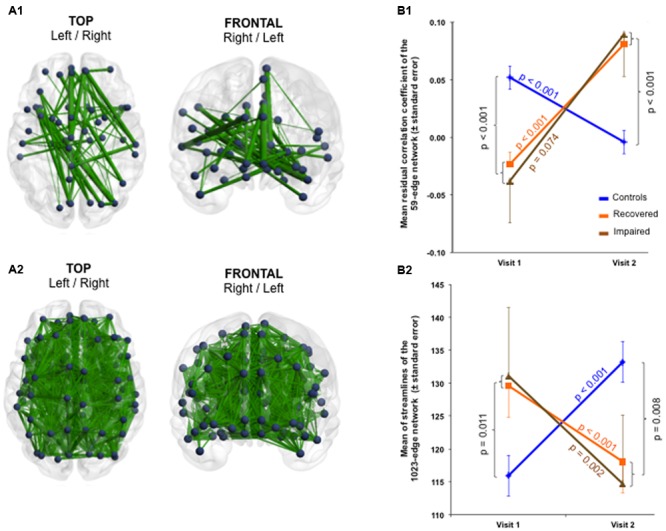
Functional and structural large-scale changes over 1 year (whole-brain group × time interaction). The NBS-specific set threshold for the functional connectivity was set to *t* = 2.42, while that of the structural connectivity was set to *t* = 0.65. **(A1)** A functional subnetwork composed of 59 edges and 48 nodes was detected (Cohen’s *d* = 1.87, 95% CI = 1.402–2.353, *p* = 0.045, corrected for multiple comparisons). **(A2)** A widespread structural subnetwork composed of 1,023 edges and 90 nodes was found (Cohen’s *d* = –1.85, 95% CI = –2.325 to –1.378, *p* = 0.045, corrected). **(B1)** At the large-scale level, functional connectivity increased in mTBI patients over time, **(B2)** whereas structural connectivity decreased. The *p*-values in the figure refer to the group effect of the patients without chronic post-concussive syndrome plotted against the healthy controls. More details about the connections of the functional subnetwork can be found in Supplementary Table 6. Due to the large number of connections affected, no table is provided for the structural subnetwork.

Finally, whole-brain group × time interactions for functional and structural connectivity were investigated. The functional subnetwork detected consisted of 59 edges distributed over 48 nodes and the structural subnetwork encompassed 1,023 edges distributed over all 90 nodes (**Figure 5** and Supplementary Table 6).

Although with more nodes and connections, similar scenarios as for the selective interactions were observed for the whole-brain interaction: functional connectivity increased (Cohen’s *d* = 1.87, 95% CI = 1.402–2.353, *p* = 0.045) while structural connectivity decreased within the patients over time (Cohen’s *d* = -1.85, 95% CI = -2.325 to -1.378, *p* = 0.045). In contrast to the partial recoveries of the functional and structural connections found in the selective interactions, whole-brain interactions revealed a steeper recovery trajectory in the patients (functional and structural time effect *p* < 0.001) independent of the severity of symptoms at Visit 2 (**Figure 5**). Neither the structural nor the functional connectivity alterations revealed by the selective as well as whole-brain interactions were statistically significantly correlated with age, for both across and within groups.

### Function–structure overlapping

**FIGURE 6:**
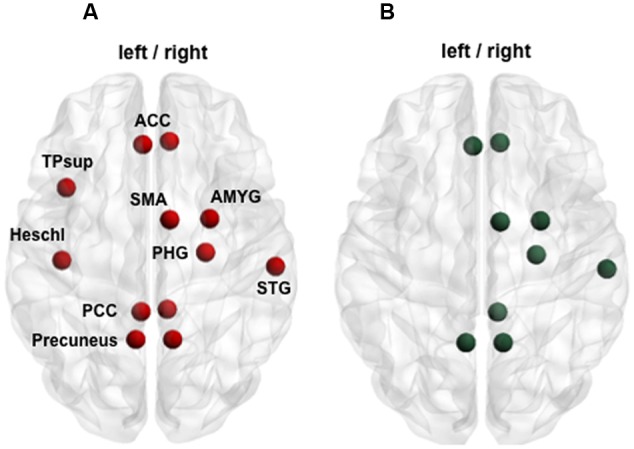
Function–structure overlapping. **(A)** Red points illustrate nodes impaired acutely after mTBI in both functional and structural connectivity analysis (group comparison at Visit 1). **(B)** Green points display partially recovered nodes over time at both functional and structural level (selective group × time interaction). ACC, anterior cingulate cortex; AMYG, amygdale; PCC, posterior cingulate cortex; PHG, parahippocampal gyrus; SMA, supplementary motor area; STG, superior temporal gyrus; TPsup, superior temporal pole.

Looking at the anatomical patterns of the brain subnetworks described above, lots of impaired but also recovered nodes of the functional and structural connectivity analyses were exactly the same. At Visit 1, the common damaged nodes encompassed bilateral ACC and PCC, precuneus, right STG, right SMA, right parahippocampal gyrus, right amygdala as well as left Heschl’s gyrus and left TP. Of these, all structures except for left PCC, left Heschl’s gyrus and left STG were also involved in the post-injury recovery phase (**Figure 6**).

Informed by functional hypoconnectivity and structural hyperconnectivity in the patients at Visit 1 and in accordance with observations from studies with moderate/severe TBI that detected more white matter disruption in patients with less functional connectivity within the DMN (Sharp et al., 2011), we hypothesized a relationship between the structurally and functionally impaired subnetworks. Indeed, we found a negative correlation between the strength of the functional with that of the structural subnetwork at Visit 1 (*r* = -0.243, *p* = 0.046, one-sided) in the group of mTBI patients, but not in controls (*r* = -0.088, *p* = 0.547). Hence we observed that the stronger the hypoconnectivity in the functional circuits the stronger the hyperconnectivity in the structural circuits.

Finally, we tested the possibility that functional connectivity reorganizations over time were affected by underlying structural reorganizations by means of restoration of diffuse axonal injuries. No significant correlations were found between longitudinal changes in the functional network with those in the structural network, neither for the selective nor for the whole-brain interaction analyses.

### Relationship between Network and Cognition Over Time

**FIGURE 7:**
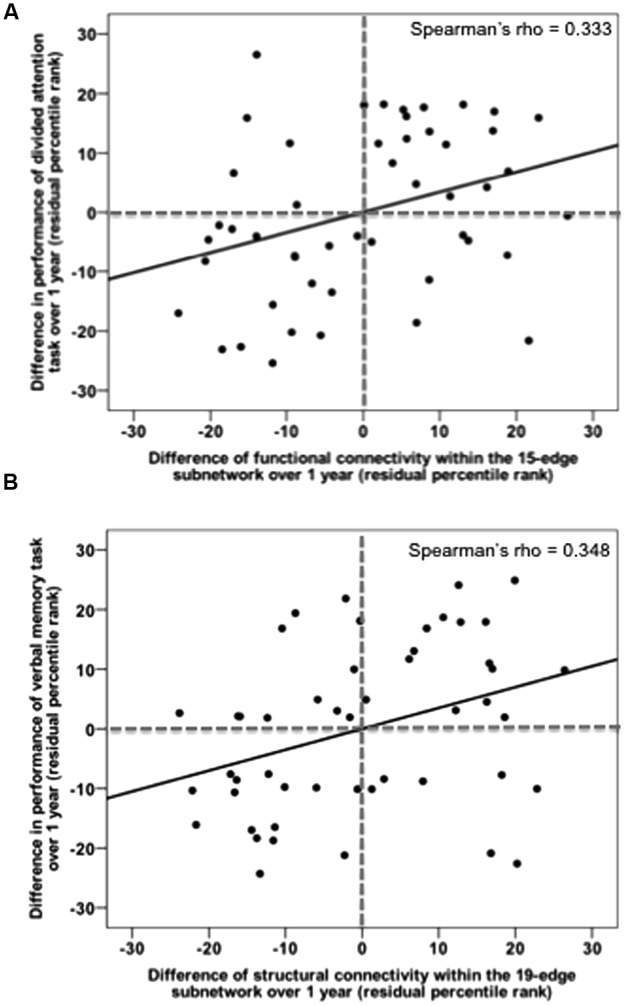
Recovery in functional and structural connectivity of the initially impaired subnetworks correlates with task improvement over 1 year post-injury. **(A)** Changes in mean functional connectivity within the default mode-like 15-edge subnetwork are plotted against changes in median reaction time on the divided attention task. **(B)** Changes in mean structural connectivity within the 19-edge subnetwork are plotted against changes in recalled items on the verbal memory task.

Relationships between alterations in mean connectivity (functional and structural) and in cognitive performance across time points were assessed within the mTBI sample only. Here, the results are reported one-sided since we expected that long-term alterations in connectivity would be associated with the—in the literature widely reported—cognitive improvement over time. We found significant correlations between functional recovery and performance improvement in a working memory test (rho = -0.350, *p* = 0.008) as well as in the speed of a divided attention task of visual stimuli (rho = 0.333, *p* = 0.012, **Figure 7A**). Decrease in functional connectivity strength of the 15-edge network, reflecting the ability to deactivate this DMN-like network, was associated with improvement in the performances of two executive tasks.

In addition, we identified a positive correlation between structural connectivity changes and improvement in a long-delayed recall test of verbal memory (rho = 0.348, *p* = 0.009, **Figure 7B**) as well as in a divided attention task (rho = -0.262, *p* = 0.039). The observed correlations were, however, not corrected for multiple testing. After the FDR adjustment tested across 30 correlations [product of 10 test scores and (i) the recovered functional subnetwork as well as recovered structural subnetworks 1 (ii) and 2 (iii), respectively], uncorrected significances did not survive the FDR correction (Supplementary Table 7). No correlations were found in patients neither for any other neuropsychological assessments nor for the clinical measurements.

## Discussion

The present study tracks, for the first time to the best of our knowledge, large-scale dynamics of resting-state functional and DTI-based structural connectivity over a period of 1-year following mTBI in a relatively large sample of 49 mTBI patients and 49 controls. We revealed three main findings: (i) The acute scenario of the injured brain started with functional hypoconnectivity in a subnetwork broadly analogous to the classical DMN and structural hyperconnectivity in a subnetwork involving widespread brain areas. The initially impaired functional and structural architectures were not only inversely related to each other, but also revealed a considerable anatomical overlap. (ii) Longitudinally, we demonstrated a partial recovery of both subnetworks disturbed at Visit 1, along with additional, considerable compensation of functional and structural connectivity patterns altered subsequent to Visit 1. (iii) We provided evidence that connectivity changes over time were clinically relevant.

### Alterations in Functional and Structural Connectivity in the Acute Phase

Considering the lack of consensus about the location of mTBI-induced brain alterations and the distributed effect of diffuse axonal injuries on the brain, we employed a whole-brain rather than a seed-based hypothesis-driven approach.

#### Functional Connectivity

Firstly, the functional analysis detected a subnetwork of reduced connectivity in patients composed of 15 connections and 15 nodes. Of these, 11 nodes are known to be part of the DMN, namely bilateral PCC, ACC, precuneus, STG, TP, and the right parahippocampal gyrus (Greicius et al., 2003; Buckner et al., 2008). Furthermore, this subnetwork, composed of mostly inter-hemispheric connections, exhibited four additional nodes arranged outside the DMN components, i.e., bilateral Heschl’s gyrus, right SMA and right amygdala. The pattern of this DM-like subnetwork differed from the pattern of the classical DMN, particularly due to the absence of the medial PFC that, together with the PCC, reflect the core set of hubs within the DMN (Andrews-Hanna et al., 2010). However, our findings are in accordance with previous mTBI studies that also observed a shift of the DMN toward functional hypoconnectivity within 7 days (Iraji et al., 2015; Zhu et al., 2015), within 11 days (Sours et al., 2015b) and in the semi-acute stage post-injury (Mayer et al., 2011; Johnson et al., 2012). Some of these studies did not focus exclusively on connectivity within the DMN and also demonstrated increased connectivity between DMN and task-positive networks, or among other brain regions (Mayer et al., 2011; Iraji et al., 2015). One reason why our results did not reveal functional hyperconnectivity between multiple networks may arise from the different method used to determine the network of interest (ROI-based versus whole-brain approach). However, other studies examining whole-brain functional connectivity by means of independent component analysis, which is not biased by a priori assumptions, detected profiles of decreased as well as increased connectivity in the same sample of semi-acutely injured mTBI patients (Shumskaya et al., 2012; Stevens et al., 2012).

#### Structural Connectivity

Secondly, the structural analysis showed increased connectivity for patients in a 53-edge subnetwork composed of mainly intra-hemispheric connections and bilateral structures. In part, these structures were already detected in the functional connectivity analysis, including bilateral ACC, PCC, precuneus, STG, TP, and right parahippocampal gyrus. The 53-edge subnetwork revealed increased connectivity of central hub areas comprising superior frontal cortex, superior parietal cortex, precuneus and subcortical putamen, thalamus, and hippocampus. These specific hub regions were found to be more densely connected among themselves than to other regions of the human connectome, suggesting a “rich-club” organization (van den Heuvel and Sporns, 2011). Highly connected central hubs of the brain are known to be vulnerable targets susceptible to disturbance in neurological disorders such as Alzheimer’s disease (Stam et al., 2009) as well as fundamental for multiple cognitive functions (van den Heuvel et al., 2009b). Surprisingly, our findings detected alterations in these densely connected network hubs, for example, the PCC and ACC, and interestingly it has been reported that these are affected in moderate/severe TBI too (Sharp et al., 2011, 2014; Pandit et al., 2013). This suggests an analogy between the pathomechanisms of mTBI and that of TBI. Stam considered the selective vulnerability of hub regions with the mechanism of “hub overload” followed by “hub failure” (Stam, 2014). In his model, the healthy neuronal network is illustrated as a hierarchical tree, where nodes at the lowest level could represent primary sensory and motor regions, while hubs at subsequent higher levels may represent multi- or supramodal association areas. Disruption of some nodes diminished their ability to handle incoming information, resulting in information traffic being rerouted to nodes higher up in the hierarchy. As a result, the traffic load of higher nodes increases and is redirected again to nodes even higher up, until the highest hubs are reached. This mechanism provokes a hub overload (Stam, 2014). Our findings of increased structural connectivity in the acute phase can therefore be explained with a rerouting of damaged nodes to the highest hubs. To directly compare our data with studies reporting traditional DTI parameters, we also explored FA values within the affected subnetwork. The increased number of streamlines reflected increased FA and this is further supported by a strong positive correlation between the two measures. In the extensive DTI literature and as recently reviewed, higher FA is not uncommon when focusing on the acute mTBI stage (Dodd et al., 2014; Eierud et al., 2014; Pacifico et al., 2015).

#### Functional–structural association

Next, we observed a functional–structural relationship between the altered connectivity patterns. The functionally hypoconnected and the structurally hyperconnected subnetwork displayed 12 nodes, but no connections in common. At first glance, these two findings might appear contradictory. On the one hand, there is no function–structure overlap at the connection level, leading to the interpretation that there is no association between disturbed functional and structural connections. However, we believe that this alternative explanation is unlikely; in fact our data also showed that functional connectivity reductions were associated with structural connectivity enhancements. In addition, the reduction of the DM-like subnetwork could be explained by indirect anatomical connections linked through a third-party region (Honey et al., 2009). Nevertheless, these 12 nodes within the DM-like subnetwork were one-to-one mirrored by the underlying impaired structural network. Numerous studies in healthy subjects have demonstrated that structural and functional resting-state connectivity are strongly interrelated, especially at the DMN level (Hagmann et al., 2008; Greicius et al., 2009; Honey et al., 2009; van den Heuvel et al., 2009a). Collectively, decreased functional connectivity within the DMN coupled with increased structural connectivity between highly central hubs might be used as a double biomarker in acute mTBI.

### Recovery of Functional and Structural Connectivity

Overall, the group × time interaction resulted in a normalization of the connections of the patients over the year, characterized by an increase in functional connectivity and a decrease in structural connectivity (as well as in mean FA). Nevertheless, the compensatory reorganization of both functional and structural subnetworks was not the same for the different interaction approaches. The selective interaction that tracked the impaired functional and structural subnetworks found at Visit 1 reached only a partial normalization, in fact, differences between the groups weakened over time, but did not completely disappear until Visit 2. In contrast, the whole-brain interaction revealed a stronger restoration, marked by pronounced increases in functional connectivity and pronounced decreases in structural connectivity of the patients involving numerous nodes and connections. We attributed these diverse longitudinal scenarios to the quite dissimilar connections analyzed. In fact, the selective interaction only accounted for connections damaged within the first 7 days, whereas the whole-brain interaction accounted for connections affected later in the course of mTBI. For example, only two functional connections were shared by the 15-edge and 59-edge subnetwork resulted from the selective and the whole-brain approach, respectively. The prominent whole-brain compensation might be attributable to the local recruitment of new structural connections. The evolution of mTBI after the acute stage may reflect a transfer from global to more local brain communication, since the brain probably starts to reroute information traffic to nodes at a lower order to alleviate the hub overload of the highest nodes in the hierarchy (Stam, 2014). As months pass by, this strong local reorganization might initiate the normalization, leading to a more efficient balance between local and global information flow. The use of two complementary interaction approaches showed that different brain regions need different times to recover. Highly interconnected hubs of the connectome seem to be the hardest to fully recover after mTBI. In the literature, connectivity studies with a follow-up of 1 year after mTBI are challenging to find. Nevertheless, our results are consistent with prior prospective studies that reported partial recovery in functional connectivity after 6 months (Bharath et al., 2015; Sours et al., 2015a) and in structural diffusion metrics (decrease in FA) after 3–5 months (Mayer A. R. et al., 2010; Ling et al., 2012). Other studies failed to detect longitudinal changes in resting-state functional connectivity during a 4-month (Mayer et al., 2011) and a 6-month period (Sours et al., 2015b). A critical distinction with previous studies that exclusively assessed symptomatic patients in the chronic stage is further worthwhile, although the findings in this subset of patients may not be generalized to the entire mTBI population. Using rsfMRI data, pronounced decrease in graph properties in frontal regions were found in mTBI patients with persistent post-concussion syndrome at the late phase in comparison to patients without complaints (Messé et al., 2013).

By means of DTI, there is a growing consensus that FA is decreased in the chronic phase of symptomatic mTBI, especially in the corpus callosum, fornix, anterior corona radiata, uncinate fasciculus, inferior longitudinal fasciculus, and in the cingulum (Niogi et al., 2008; Niogi and Mukherjee, 2010; Eierud et al., 2014; Dean et al., 2015). Increased mean diffusivity in similar long association tracts as reported above has also been found in relation to poor outcome showing that those patients had greater and wider structural damages at late phase than patients with good outcome (Messé et al., 2011, 2012).

Although the duration of a full recovery is not yet clear, the current study suggests that neuroplasticity after mTBI necessitates more than 1 year to completely restore, both for structural and functional networks. As already reported in other studies, our findings are in agreement that residue of physiological anomalies are difficult to detect with standard clinical and cognitive assessments, as these symptoms were mostly already returned to baseline (Zhu et al., 2015). Finally, the degree to which network recovery differs between patients with and without persistent symptoms is under debate. At the functional level, the comparison between patients with and without PCD revealed no differences at Visit 2, suggesting decoupling between compensatory brain response to mTBI and clinical symptomatology. The structural recovery was analogous for symptomatic and asymptomatic patients when looking at the whole-brain recovery, but indicated a slight trend toward distinctive courses when investigating the initially impaired subnetwork longitudinally. However, here only a handful of patients (*n* = 6) demonstrated PCD after 1 year and therefore a larger sample size is required to verify our findings. Other factors indirectly related to mTBI such as insomnia, fatigue, post-traumatic headache, pre-injury problems, or psychological distress might play a role in the maintenance of protracted symptoms after mTBI (Zumstein et al., 2011; Silverberg et al., 2015).

### Relationship between Network and Cognition over Time

Some mTBI studies have supported the relationship between an anomal pattern of connectivity and poorer cognitive performance, especially in attention, executive function, and working memory (Mayer et al., 2015; Dall’Acqua et al., 2016). Only few studies looked at the correlation between changes in the brain and changes in cognition over a long time period (Croall et al., 2014). Above, we showed a longitudinal increase in functional connectivity within the DM-like subnetwork in patients interpreted as reflecting recovery, which therefore may be linked to cognitive improvement. Unexpectedly, we found that the lower the functional connectivity of the DM-like subnetwork the better the cognitive performance in a working memory and divided attention task. This correlation should be viewed in the context of the ability of the “recovered” DMN to attenuate its activity during goal-focused tasks (Greicius et al., 2003). The DMN is a network of functionally connected structures synchronously activated at rest and during internally directed processes, but synchronously deactivated during external task conditions (Raichle et al., 2001). The DMN has an essential role in cognitive functions and task-evoked activity is intimately related to functional connectivity identified in the resting brain (Smith et al., 2009; Laird et al., 2011). Failure to deactivate the DMN, in particular the PCC as one of its core nodes, has been associated with poor cognitive performance in mTBI and TBI, but also in healthy subjects (Weissman et al., 2006; Leech and Sharp, 2014; van der Horn et al., 2015). Attenuation of DMN activity in order to allow the task-positive networks to be activated has been described as load-dependent: the greater the cognitive effort required from the task the stronger the inhibition of the DMN (Mayer J. S. et al., 2010). In line with these observations, we hypothesized that patients able to robustly inhibit DMN activity performed better in cognitively high demanding tasks such as working memory and divided attention. This interpretation has not been explicitly tested in our study because our patients did not actively participate in an fMRI experiment that would allow a direct comparison between their brain activation and their behavioral responses.

Finally, the finding that decreased structural connectivity was related to verbal memory and divided attention worsening over time was puzzling due to its apparent contradiction. While speculative, we interpreted the decrease in structural connectivity (measured by both the number of streamlines as well as FA) as recovery mechanism since the majority of the patients recovered over time. On the contrary, the literature indicated that low FA findings are more frequently reported in association with poor neuropsychological performance in studies of chronic mTBI (Eierud et al., 2014). Nevertheless, the link between clinical outcomes and the direction of the FA change remain controversial and the authors of the meta-analysis also suggested that factors contributing to increased FA in the acute phase, i.e., increased intracellular and decreased extracellular water within the myelin sheath, are no longer valid in the chronic phase.

This idea is in line with our findings, since the longitudinal decrease in the number of streamlines over the year corresponded to a decrease in FA. After 1 year, the premorbid status, in which high structural connectivity leads to better performance, was restored again (Tamnes et al., 2011). Interpretations of the abovementioned correlations have to be done with caution since they were uncorrected for multiple testing.

## Limitations and Conclusion

The present study has some limitations that are worth mentioning. To track the recovery until exhaustion of its potential, future longitudinal studies should scan beyond 12 months. The small number of mTBI patients (*n* = 6) having a PCD in the chronic phase (∼12%) may affect robust interpretations and compromises the overall generalizability of the results. Even though this percentage is consistent with the mTBI literature (Hou et al., 2012), a larger sample size with symptomatic patients is required to improve the power of the statistical analysis. In addition, it should be considered that the patient group consisted of a convenience sample recruited from four different emergency department according to the availability of the study team and may not be representative of the whole mTBI population. At the same time, the study took advantage of the low long-term dropout rate, motivated through various incentives including financial compensation, free transport service and intensive contact strategy.

Finally, functional and structural alterations of the healthy subjects over time were somewhat unexpected and have the potential to complicate our understanding of the group × time interaction. Studies examining the long-term test–retest reliability of graph metrics derived from rsfMRI data have documented heterogeneous results, spanning from low to high network reliability in young adults (Wang et al., 2011; Franco et al., 2013; Du et al., 2015). These results are likely to be dependent on multiple factors such as the network identification analysis (whole-brain, seed-based or independent-component analysis) and the definition of long-term reliability. Even so, other evidence suggests that dynamic changes in resting-state networks might emerge depending on the subjective mental state of participants during scanning (Waites et al., 2005; Newton et al., 2007). A study using a mood-induction paradigm found increased functional connectivity with increasing subjective experience of sadness in a paralimbic network (Harrison et al., 2008). These findings fit well with the functional hyperconnectivity observed in the DM-like subnetwork of our controls at Visit 1 involving bilateral ACC and SMA. Collectively, low test–retest reliability in our controls might originate from the emotional state related to the first scan (e.g., anxiety, tension) that was no longer present at the second scan. In contrast, excessive fatigue and drowsiness of the patients due to their recent mTBI may have influenced their dominant subjective state at Visit 1, rather than any emotional involvement. Finally, change in functional connectivity within the HC has been previously described in a TBI study (Hillary et al., 2011). Here—as in our study—the HC showed a decrease in functional connectivity, whereas the patients showed an increase between time points (3 months interval).

Studies on long-term test–retest reliability of network measurements derived from DTI data throughout adulthood are still lacking. However, a study examining diffusion MRI during late adolescence over a 2-year period suggested increased structural connectivity between frontal and subcortical hubs (Baker et al., 2015). These authors reported evidence of selective refinement of connections in healthy adolescents over time and this maturational process might support the increase in structural connectivity over the year in our control sample. Similar observations were made in a recent DTI study in schizophrenia that employed graph theoretical analysis over a 5-year period and found an increase of nodal efficiency (global integration) in the healthy controls suggesting maturational and/or plasticity-related processes in the network as well as in a cross sectional study examining the structural connectome of healthy individual between 12 and 30 years that exhibited increased network integration (Dennis et al., 2013; Sun et al., 2016). Lastly, confounding sources not completely controllable affecting intrasession DTI variability include subject head motion (Jones and Cercignani, 2010; Wang et al., 2012; Jones et al., 2013). We therefore investigated levels of head motion in our healthy controls between visits, but did not find any significant translational or rotational head motions changes (average volume-by-volume translations *p* = 0.618 and average volume-by-volume rotations *p* = 0.965). We could so ensure that translational and rotational motions were comparable between Visit 1 and 2.

Our DTI analysis is based on standard procedures widely used in clinical settings that, however, have also their shortcomings. Although the single tensor model cannot deal with voxels containing crossing fibers, this limitation is present in both of our groups at both time points and hence should not bias the group comparisons and interactions reported. We recognize that there are fundamental limitations on the ability of tensor-based tractography to accurately estimate complex fiber configurations and that the use of higher-order tractography models on diffusion-weighted data such as constrained spherical deconvolution or automatic relevance determination would be definitively more favorable (Behrens et al., 2007; Tournier et al., 2007). These more sophisticated reconstruction algorithms clearly demonstrated fiber tracts more accurately in the presence of multiple fiber populations within a voxel (Farquharson et al., 2013; Jeurissen et al., 2013). Therefore, the results derived from DTI need to be interpreted with caution. For future diffusion imaging projects, we considered to apply superior diffusion models for the downstream processing as well as newer analysis tools such as MRtrix3.

Finally, linear “eddy_correct” to correct for eddy current-induced off-resonance field is widely used in clinical settings. Although we found equivalent effect on the structural connectome across the different corrections (eddy and eddy_correct), recent publications revealed that this model is insufficient for diffusion data measured with high-*b*-values and that a higher order model performs better (Yamada et al., 2014; Andersson and Sotiropoulos, 2016). Future diffusion imaging studies should employ the eddy and topup tools, which offer superior correction for distortion and will provide more accurate insights into the structural network alterations following mTBI.

In conclusion, the current study demonstrates the involvement of networks similar to the DMN and of central hub areas in the pathophysiology of mTBI. Functional and structural compensatory processes differ between brain regions with respect to their time course and are not completed after 1 year, in particular when central hubs are involved. It remains unknown if the impaired functional and structural network connectivity will ever reach the premorbid level. This multimodal study highlights for the first time the importance of scanning the brain over a longer period than 1 year post-injury. Future longitudinal investigations should extend the time horizon to track down the full dynamics of neuronal plasticity, which could be used by clinicians to update their management, intervention and prevention after a single or repeated mTBI.

## Author Contributions

PD and JH contributed to the design of the study, to acquisition, analysis, interpretation of data, and drafting the manuscript. SJ contributed to the design and conception of the study, interpretation of data, and critically revised the manuscript. LM, H-PS, RG, JF, MS, and CM contributed to data acquisition and critically revised the manuscript. EU analyzed the neuroradiological data and critically revised the manuscript. AM contributed to the design and conception of the study and critically revised the manuscript. HB contributed to data analysis and critically revised the manuscript. LJ contributed to the design of the study, interpretation of data, and critically revised the manuscript. All authors are accountable for all aspects of the work and approved the final version of the manuscript to be published.

## Conflict of Interest Statement

PD and SJ are employed by SUVA. SJ, LM, H-PS, RG, JF, MS, CM, EU, AM, HB, and LJ report no disclosures relevant to the manuscript. LJ has received other research support from SUVA. The other author declares that the research was conducted in the absence of any commercial or financial relationships that could be construed as a potential conflict of interest.

## References

[B1] Andersson J. L. Sotiropoulos S. N. (2016). An integrated approach to correction for off-resonance effects and subject movement in diffusion MR imaging. *Neuroimage* 125 1063–1078. 10.1016/j.neuroimage.2015.10.019 26481672 PMC4692656

[B2] Andrews-Hanna J. R. Reidler J. S. Sepulcre J. Poulin R. Buckner R. L. (2010). Functional-anatomic fractionation of the brain’s default network. *Neuron* 65 550–562. 10.1016/j.neuron.2010.02.005 20188659 PMC2848443

[B3] Baker S. T. Lubman D. I. Yucel M. Allen N. B. Whittle S. Fulcher B. D. (2015). Developmental changes in brain network hub connectivity in late adolescence. *J. Neurosci.* 35 9078–9087. 10.1523/JNEUROSCI.5043-14.2015 26085632 PMC6605159

[B4] Behrens T. E. Berg H. J. Jbabdi S. Rushworth M. F. Woolrich M. W. (2007). Probabilistic diffusion tractography with multiple fibre orientations: what can we gain? *Neuroimage* 34 144–155. 10.1016/j.neuroimage.2006.09.018 17070705 PMC7116582

[B5] Bharath R. D. Munivenkatappa A. Gohel S. Panda R. Saini J. Rajeswaran J. (2015). Recovery of resting brain connectivity ensuing mild traumatic brain injury. *Front. Hum. Neurosci.* 9:513 10.3389/fnhum.2015.00513 PMC4585122 26441610

[B6] Buckner R. L. Andrews-Hanna J. R. Schacter D. L. (2008). The brain’s default network: anatomy, function, and relevance to disease. *Ann. N. Y. Acad. Sci.* 1124 1–38. 10.1196/annals.1440.011 18400922

[B7] Bullmore E. Sporns O. (2009). Complex brain networks: graph theoretical analysis of structural and functional systems. *Nat. Rev. Neurosci.* 10 186–198. 10.1038/nrn2575 19190637

[B8] Cassidy J. D. Carroll L. J. Peloso P. M. Borg J. von Holst H. Holm L. (2004). Incidence, risk factors and prevention of mild traumatic brain injury: results of the WHO Collaborating Centre Task Force on Mild Traumatic Brain Injury. *J. Rehabil. Med.* 43(Suppl. 43) 28–60. 10.1080/16501960410023732 15083870

[B9] Cohen J. (1988). *Statistical Power Analysis for the Behavioral Sciences* 2nd Edn. Hillsdale, NJ: Erlbaum.

[B10] Croall I. D. Cowie C. J. He J. Peel A. Wood J. Aribisala B. S. (2014). White matter correlates of cognitive dysfunction after mild traumatic brain injury. *Neurology* 83 494–501. 10.1212/WNL.0000000000000666 25031282 PMC4142001

[B11] Dall’Acqua P. Johannes S. Mica L. Simmen H. P. Glaab R. Fandino J. (2016). Connectomic and surface-based morphometric correlates of acute mild traumatic brain injury. *Front. Hum. Neurosci.* 10:127 10.3389/fnhum.2016.00127 PMC4809899 27065831

[B12] Dean P. J. A. Sato J. R. Vieira G. McNamara A. Sterr A. (2015). Long-term structural changes after mTBI and their relation to post-concussion symptoms. *Brain Inj.* 29 1211–1218. 10.3109/02699052.2015.1035334 26067623

[B13] Dennis E. L. Jahanshad N. McMahon K. L. de Zubicaray G. I. Martin N. G. Hickie I. B. (2013). Development of brain structural connectivity between ages 12 and 30: a 4-Tesla diffusion imaging study in 439 adolescents and adults. *Neuroimage* 64 671–684. 10.1016/j.neuroimage.2012.09.004 22982357 PMC3603574

[B14] Dodd A. B. Epstein K. Ling J. Mayer A. (2014). Diffusion tensor imaging findings in semi-acute mild traumatic brain injury. *J. Neurotrauma* 31 1235–1248. 10.1089/neu.2014.3337 24779720

[B15] Du H. X. Liao X. H. Lin Q. X. Li G. S. Chi Y. Z. Liu X. (2015). Test-retest reliability of graph metrics in high-resolution functional connectomics: a resting-state functional MRI study. *CNS Neurosci. Ther.* 21 802–816. 10.1111/cns.12431 26212146 PMC6493187

[B16] Eierud C. Craddock R. C. Fletcher S. Aulakh M. King-Casas B. Kuehl D. (2014). Neuroimaging after mild traumatic brain injury: review and meta-analysis. *Neuroimage Clin.* 4 283–294. 10.1016/j.nicl.2013.12.009 25061565 PMC4107372

[B17] Farquharson S. Tournier J. D. Calamante F. Fabinyi G. Schneider-Kolsky M. Jackson G. D. (2013). White matter fiber tractography: why we need to move beyond DTI. *J. Neurosurg.* 118 1367–1377. 10.3171/2013.2.JNS121294 23540269

[B18] Franco A. R. Mannell M. V. Calhoun V. D. Mayer A. R. (2013). Impact of analysis methods on the reproducibility and reliability of resting-state networks. *Brain Connect.* 3 363–374. 10.1089/brain.2012.0134 23705789 PMC3749744

[B19] Greicius M. D. Krasnow B. Reiss A. L. Menon V. (2003). Functional connectivity in the resting brain: a network analysis of the default mode hypothesis. *Proc. Natl. Acad. Sci. U.S.A.* 100 253–258. 10.1073/pnas.0135058100 12506194 PMC140943

[B20] Greicius M. D. Supekar K. Menon V. Dougherty R. F. (2009). Resting-state functional connectivity reflects structural connectivity in the default mode network. *Cereb. Cortex* 19 72–78. 10.1093/cercor/bhn059 18403396 PMC2605172

[B21] Hagmann P. Cammoun L. Gigandet X. Meuli R. Honey C. J. Wedeen V. J. (2008). Mapping the structural core of human cerebral cortex. *PLoS Biol.* 6:e159 10.1371/journal.pbio.0060159 PMC2443193 18597554

[B22] Harrison B. J. Pujol J. Ortiz H. Fornito A. Pantelis C. Yucel M. (2008). Modulation of brain resting-state networks by sad mood induction. *PLoS ONE* 3:e1794 10.1371/journal.pone.0001794 PMC2263138 18350136

[B23] Hillary F. G. Slocomb J. Hills E. C. Fitzpatrick N. M. Medaglia J. D. Wang J. (2011). Changes in resting connectivity during recovery from severe traumatic brain injury. *Int. J. Psychophysiol.* 82 115–123. 10.1016/j.ijpsycho.2011.03.011 21473890

[B24] Honey C. J. Sporns O. Cammoun L. Gigandet X. Thiran J. P. Meuli R. (2009). Predicting human resting-state functional connectivity from structural connectivity. *Proc. Natl. Acad. Sci. U.S.A.* 106 2035–2040. 10.1073/pnas.0811168106 19188601 PMC2634800

[B25] Hou R. Moss-Morris R. Peveler R. Mogg K. Bradley B. P. Belli A. (2012). When a minor head injury results in enduring symptoms: a prospective investigation of risk factors for postconcussional syndrome after mild traumatic brain injury. *J. Neurol. Neurosurg. Psychiatry* 83 217–223. 10.1136/jnnp-2011-300767 22028384

[B26] Iraji A. Benson R. R. Welch R. D. O’Neil B. J. Woodard J. L. Ayaz S. I. (2015). Resting state functional connectivity in mild traumatic brain injury at the acute stage: independent component and seed-based analyses. *J. Neurotrauma* 32 1031–1045. 10.1089/neu.2014.3610 25285363 PMC4504339

[B27] Jeurissen B. Leemans A. Tournier J. D. Jones D. K. Sijbers J. (2013). Investigating the prevalence of complex fiber configurations in white matter tissue with diffusion magnetic resonance imaging. *Hum. Brain Mapp.* 34 2747–2766. 10.1002/hbm.22099 22611035 PMC6870534

[B28] Johnson B. Zhang K. Gay M. Horovitz S. Hallett M. Sebastianelli W. (2012). Alteration of brain default network in subacute phase of injury in concussed individuals: resting-state fMRI study. *Neuroimage* 59 511–518. 10.1016/j.neuroimage.2011.07.081 21846504 PMC3196274

[B29] Jones D. K. Cercignani M. (2010). Twenty-five pitfalls in the analysis of diffusion MRI data. *NMR Biomed.* 23 803–820. 10.1002/nbm.1543 20886566

[B30] Jones D. K. Knosche T. R. Turner R. (2013). White matter integrity, fiber count, and other fallacies: the do’s and don’ts of diffusion MRI. *Neuroimage* 73 239–254. 10.1016/j.neuroimage.2012.06.081 22846632

[B31] King N. S. Crawford S. Wenden F. J. Moss N. E. Wade D. T. (1995). The Rivermead Post Concussion Symptoms Questionnaire: a measure of symptoms commonly experienced after head injury and its reliability. *J. Neurol.* 242 587–592. 10.1007/BF00868811 8551320

[B32] Laird A. R. Fox P. M. Eickhoff S. B. Turner J. A. Ray K. L. McKay D. R. (2011). Behavioral interpretations of intrinsic connectivity networks. *J. Cogn. Neurosci.* 23 4022–4037. 10.1162/jocn_a_00077 21671731 PMC3690655

[B33] Leech R. Sharp D. J. (2014). The role of the posterior cingulate cortex in cognition and disease. *Brain* 137(Pt 1) 12–32. 10.1093/brain/awt162 23869106 PMC3891440

[B34] Ling J. M. Pena A. Yeo R. A. Merideth F. L. Klimaj S. Gasparovic C. (2012). Biomarkers of increased diffusion anisotropy in semi-acute mild traumatic brain injury: a longitudinal perspective. *Brain* 135(Pt 4) 1281–1292. 10.1093/brain/aws073 22505633 PMC3326260

[B35] Mayer A. R. Bellgowan P. S. Hanlon F. M. (2015). Functional magnetic resonance imaging of mild traumatic brain injury. *Neurosci. Biobehav. Rev.* 49 8–18. 10.1016/j.neubiorev.2014.11.016 25434880

[B36] Mayer A. R. Ling J. Mannell M. V. Gasparovic C. Phillips J. P. Doezema D. (2010). A prospective diffusion tensor imaging study in mild traumatic brain injury. *Neurology* 74 643–650. 10.1212/WNL.0b013e3181d0ccdd 20089939 PMC2830922

[B37] Mayer A. R. Mannell M. V. Ling J. Gasparovic C. Yeo R. A. (2011). Functional connectivity in mild traumatic brain injury. *Hum. Brain Mapp.* 32 1825–1835. 10.1002/hbm.21151 21259381 PMC3204375

[B38] Mayer J. S. Roebroeck A. Maurer K. Linden D. E. (2010). Specialization in the default mode: task-induced brain deactivations dissociate between visual working memory and attention. *Hum. Brain Mapp.* 31 126–139. 10.1002/hbm.20850 19639552 PMC6870780

[B39] Messé A. Caplain S. Paradot G. Garrigue D. Mineo J. F. Soto Ares G. (2011). Diffusion tensor imaging and white matter lesions at the subacute stage in mild traumatic brain injury with persistent neurobehavioral impairment. *Hum. Brain Mapp.* 32 999–1011. 10.1002/hbm.21092 20669166 PMC6870077

[B40] Messé A. Caplain S. Pélégrini-Issac M. Blancho S. Lévy R. Aghakhani N. (2013). Specific and evolving resting-state network alterations in post-concussion syndrome following mild traumatic brain injury. *PLoS ONE* 8:e65470 10.1371/journal.pone.0065470 PMC3675039 23755237

[B41] Messé A. Caplain S. Pélégrini-Issac M. Blancho S. Montreuil M. Lévy R. (2012). Structural integrity and postconcussion syndrome in mild traumatic brain injury patients. *Brain Imaging Behav.* 6 283–292. 10.1007/s11682-012-9159-2 22477019

[B42] Nakamura T. Hillary F. G. Biswal B. B. (2009). Resting network plasticity following brain injury. *PLoS ONE* 4:e8220 10.1371/journal.pone.0008220 PMC2788622 20011533

[B43] Newton A. T. Morgan V. L. Gore J. C. (2007). Task demand modulation of steady-state functional connectivity to primary motor cortex. *Hum. Brain Mapp.* 28 663–672. 10.1002/hbm.20294 17080441 PMC6871425

[B44] Niogi S. N. Mukherjee P. (2010). Diffusion tensor imaging of mild traumatic brain injury. *J. Head Trauma Rehabil.* 25 241–255. 10.1097/HTR.0b013e3181e52c2a 20611043

[B45] Niogi S. N. Mukherjee P. Ghajar J. Johnson C. Kolster R. A. Sarkar R. (2008). Extent of microstructural white matter injury in postconcussive syndrome correlates with impaired cognitive reaction time: A_3_T diffusion tensor imaging study of mild traumatic brain injury. *AJNR Am. J. Neuroradiol.* 29 967–973. 10.3174/ajnr.A0970 18272556 PMC8128563

[B46] Pacifico A. Amyot F. Arciniegas D. Brazaitis M. P. Curley K. Diaz-Arrastia R. (2015). A review of the effectiveness of neuroimaging modalities for the detection of traumatic brain injury. *J. Neurotrauma* 32 1693–1721. 10.1089/neu.2013.3306 26176603 PMC4651019

[B47] Pandit A. S. Expert P. Lambiotte R. Bonnelle V. Leech R. Turkheimer F. E. (2013). Traumatic brain injury impairs small-world topology. *Neurology* 80 1826–1833. 10.1212/WNL.0b013e3182929f38 23596068 PMC3908350

[B48] Power J. D. Barnes K. A. Snyder A. Z. Schlaggar B. L. Petersen S. E. (2012). Spurious but systematic correlations in functional connectivity MRI networks arise from subject motion. *Neuroimage* 59 2142–2154. 10.1016/j.neuroimage.2011.10.018 22019881 PMC3254728

[B49] Power J. D. Schlaggar B. L. Petersen S. E. (2015). Recent progress and outstanding issues in motion correction in resting state fMRI. *Neuroimage* 105 536–551. 10.1016/j.neuroimage.2014.10.044 25462692 PMC4262543

[B50] Raichle M. E. MacLeod A. M. Snyder A. Z. Powers W. J. Gusnard D. A. Shulman G. L. (2001). A default mode of brain function. *Proc. Natl. Acad. Sci. U.S.A.* 98 676–682. 10.1073/pnas.98.2.676 11209064 PMC14647

[B51] Sharp D. J. Beckmann C. F. Greenwood R. Kinnunen K. M. Bonnelle V. De Boissezon X. (2011). Default mode network functional and structural connectivity after traumatic brain injury. *Brain* 134(Pt 8) 2233–2247. 10.1093/brain/awr175 21841202

[B52] Sharp D. J. Scott G. Leech R. (2014). Network dysfunction after traumatic brain injury. *Nat. Rev. Neurol.* 10 156–166. 10.1038/nrneurol.2014.15 24514870

[B53] Shumskaya E. Andriessen T. M. Norris D. G. Vos P. E. (2012). Abnormal whole-brain functional networks in homogeneous acute mild traumatic brain injury. *Neurology* 79 175–182. 10.1212/WNL.0b013e31825f04fb 22744656

[B54] Silverberg N. D. Gardner A. J. Brubacher J. R. Panenka W. J. Li J. J. Iverson G. L. (2015). Systematic review of multivariable prognostic models for mild traumatic brain injury. *J. Neurotrauma* 32 517–526. 10.1089/neu.2014.3600 25222514

[B55] Smith S. M. Fox P. T. Miller K. L. Glahn D. C. Fox P. M. Mackay C. E. (2009). Correspondence of the brain’s functional architecture during activation and rest. *Proc. Natl. Acad. Sci. U.S.A.* 106 13040–13045. 10.1073/pnas.0905267106 19620724 PMC2722273

[B56] Sours C. Chen H. Roys S. Zhuo J. Varshney A. Gullapalli R. P. (2015a). Investigation of multiple frequency ranges using discrete wavelet decomposition of resting state functional connectivity in mild traumatic brain injury patients. *Brain Connect.* 5 442–450. 10.1089/brain.2014.0333 25808612 PMC4575515

[B57] Sours C. Zhuo J. Roys S. Shanmuganathan K. Gullapalli R. P. (2015b). Disruptions in resting state functional connectivity and cerebral blood flow in mild traumatic brain injury patients. *PLoS ONE* 10:e0134019 10.1371/journal.pone.0134019 PMC4524606 26241476

[B58] Stam C. J. (2014). Modern network science of neurological disorders. *Nat. Rev. Neurosci.* 15 683–695. 10.1038/nrn3801 25186238

[B59] Stam C. J. de Haan W. Daffertshofer A. Jones B. F. Manshanden I. van Cappellen van Walsum A. M. (2009). Graph theoretical analysis of magnetoencephalographic functional connectivity in Alzheimer’s disease. *Brain* 132(Pt 1) 213–224. 10.1093/brain/awn262 18952674

[B60] Stevens M. C. Lovejoy D. Kim J. Oakes H. Kureshi I. Witt S. T. (2012). Multiple resting state network functional connectivity abnormalities in mild traumatic brain injury. *Brain Imaging Behav.* 6 293–318. 10.1007/s11682-012-9157-4 22555821

[B61] Sun Y. Chen Y. Lee R. Bezerianos A. Collinson S. L. Sim K. (2016). Disruption of brain anatomical networks in schizophrenia: a longitudinal, diffusion tensor imaging based study. *Schizophr. Res.* 171 149–157. 10.1016/j.schres.2016.01.025 26811255

[B62] Tamnes C. K. Fjell A. M. Ostby Y. Westlye L. T. Due-Tonnessen P. Bjornerud A. (2011). The brain dynamics of intellectual development: waxing and waning white and gray matter. *Neuropsychologia* 49 3605–3611. 10.1016/j.neuropsychologia.2011.09.012 21939677

[B63] Tournier J. D. Calamante F. Connelly A. (2007). Robust determination of the fibre orientation distribution in diffusion MRI: non-negativity constrained super-resolved spherical deconvolution. *Neuroimage* 35 1459–1472. 10.1016/j.neuroimage.2007.02.016 17379540

[B64] Tzourio-Mazoyer N. Landeau B. Papathanassiou D. Crivello F. Etard O. Delcroix N. (2002). Automated anatomical labeling of activations in SPM using a macroscopic anatomical parcellation of the MNI MRI single-subject brain. *Neuroimage* 15 273–289. 10.1006/nimg.2001.0978 11771995

[B65] van den Heuvel M. P. Mandl R. C. Kahn R. S. Hulshoff Pol H. E. (2009a). Functionally linked resting-state networks reflect the underlying structural connectivity architecture of the human brain. *Hum. Brain Mapp.* 30 3127–3141. 10.1002/hbm.20737 19235882 PMC6870902

[B66] van den Heuvel M. P. Sporns O. (2011). Rich-club organization of the human connectome. *J. Neurosci.* 31 15775–15786. 10.1523/JNEUROSCI.3539-11.2011 22049421 PMC6623027

[B67] van den Heuvel M. P. Stam C. J. Kahn R. S. Hulshoff Pol H. E. (2009b). Efficiency of functional brain networks and intellectual performance. *J. Neurosci.* 29 7619–7624. 10.1523/JNEUROSCI.1443-09.2009 19515930 PMC6665421

[B68] van der Horn H. J. Liemburg E. J. Scheenen M. E. de Koning M. E. Spikman J. M. van der Naalt J. (2015). Post-concussive complaints after mild traumatic brain injury associated with altered brain networks during working memory performance. *Brain Imaging Behav.* 10 1243–1253. 10.1007/s11682-015-9489-y PMC5167217 26667033

[B69] Vos P. E. Alekseenko Y. Battistin L. Ehler E. Gerstenbrand F. Muresanu D. F. (2012). Mild traumatic brain injury. *Eur. J. Neurol.* 19 191–198. 10.1111/j.1468-1331.2011.03581.x 22260187

[B70] Waites A. B. Stanislavsky A. Abbott D. F. Jackson G. D. (2005). Effect of prior cognitive state on resting state networks measured with functional connectivity. *Hum. Brain Mapp.* 24 59–68. 10.1002/hbm.20069 15382248 PMC6871664

[B71] Wang J. H. Zuo X. N. Gohel S. Milham M. P. Biswal B. B. He Y. (2011). Graph theoretical analysis of functional brain networks: test-retest evaluation on short- and long-term resting-state functional MRI data. *PLoS ONE* 6:e21976 10.1371/journal.pone.0021976 PMC3139595 21818285

[B72] Wang J. Y. Abdi H. Bakhadirov K. Diaz-Arrastia R. Devous M. D. Sr. (2012). A comprehensive reliability assessment of quantitative diffusion tensor tractography. *Neuroimage* 60 1127–1138. 10.1016/j.neuroimage.2011.12.062 22227883 PMC3468740

[B73] Weissman D. H. Roberts K. C. Visscher K. M. Woldorff M. G. (2006). The neural bases of momentary lapses in attention. *Nat. Neurosci.* 9 971–978. 10.1038/nn1727 16767087

[B74] Xia M. Wang J. He Y. (2013). BrainNet Viewer: a network visualization tool for human brain connectomics. *PLoS ONE* 8:e68910 10.1371/journal.pone.0068910 PMC3701683 23861951

[B75] Yamada H. Abe O. Shizukuishi T. Kikuta J. Shinozaki T. Dezawa K. (2014). Efficacy of distortion correction on diffusion imaging: comparison of FSL eddy and eddy_correct using 30 and 60 directions diffusion encoding. *PLoS ONE* 9:e112411 10.1371/journal.pone.0112411 PMC4236106 25405472

[B76] Yendiki A. Koldewyn K. Kakunoori S. Kanwisher N. Fischl B. (2014). Spurious group differences due to head motion in a diffusion MRI study. *Neuroimage* 88 79–90. 10.1016/j.neuroimage.2013.11.027 24269273 PMC4029882

[B77] Zalesky A. Fornito A. Bullmore E. T. (2010). Network-based statistic: identifying differences in brain networks. *Neuroimage* 53 1197–1207. 10.1016/j.neuroimage.2010.06.041 20600983

[B78] Zhu D. C. Covassin T. Nogle S. Doyle S. Russell D. Pearson R. L. (2015). A potential biomarker in sports-related concussion: brain functional connectivity alteration of the default-mode network measured with longitudinal resting-state fMRI over thirty days. *J. Neurotrauma* 32 327–341. 10.1089/neu.2014.3413 25116397

[B79] Zumstein M. A. Moser M. Mottini M. Ott S. R. Sadowski-Cron C. Radanov B. P. (2011). Long-term outcome in patients with mild traumatic brain injury: a prospective observational study. *J. Trauma* 71 120–127. 10.1097/TA.0b013e3181f2d670 21045743

