# Supplementary material for: Functional and Structural Network Recovery after Mild Traumatic Brain Injury: A 1-Year Longitudinal Study
Source: Front Hum Neurosci. 2017 May 30;11:280. doi: 10.3389/fnhum.2017.00280 (PMC5447750; doi:10.3389/fnhum.2017.00280)
Supplement: Supplementary file 1 [file Data_Sheet_1.DOCX]

***Supplementary Material***

**Functional and structural network recovery after mild traumatic brain injury: A 1-year longitudinal study**

Patrizia Dall’Acqua^*^, Sönke Johannes, Ladislav Mica, Hans-Peter Simmen, Richard Glaab, Javier Fandino, Markus Schwendinger, Christoph Meier, Erika Jasmin Ulbrich, Andreas Müller, Hansruedi Baetschmann, Lutz Jäncke and Jürgen Hänggi

*Correspondence: Patrizia Dall’Acqua: [patrizia.dallacqua@rehabellikon.ch](mailto:patrizia.dallacqua@rehabellikon.ch)

**1 Supplementary Data**

**Supplementary Methods**

**Neuropsychological assessment**

The neuropsychological assessment focusing on the cognitive domains of attention, executive functions and memory included the following tests: (i) subtests Alertness (intrinsic and phasic), Go/Nogo, and Divided attention of the Test for Attentional Performance (TAP 2.2) ([Zimmermann and Fimm, 2002](#_ENREF_25)). These tests measure attention, inhibitory control, and cognitive flexibility with varying complexity; (ii) German version ([Von Aster et al., 2006](#_ENREF_21)) of the Backward Digit Span of the Wechsler Adult Intelligence Scale WAIS-III ([Wechsler, 1997](#_ENREF_22)) to assess verbal working memory; (iii) Swiss adaptation ([Balzer et al., 2011](#_ENREF_1)) of the Rey Auditory Verbal Learning Tests RAVLT ([Strauss et al., 2006](#_ENREF_19)) to assess verbal learning (using the total number of words recalled across five trials) and short- and long-delay verbal recall. In addition, an estimation of the nonverbal intelligence level has been obtained by using the Wiener Matrizen-Test 2 WMT-2 ([Formann et al., 2011](#_ENREF_10)), an adapted version based on Raven’s progressive matrix test ([Raven, 1958](#_ENREF_17)). The neuropsychological assessment also included a test measuring effort and symptom validity (Green’s Medical Symptom Validity Test, MSVT). The MSVT ([Green, 2004](#_ENREF_11)) is a brief automated verbal memory screening with several subtests designed to measure verbal memory and response consistency. In addition, two scales were used to assess emotional symptomatology: the German version ([Hautzinger et al., 2006](#_ENREF_12)) of the Beck Depression Inventory 2^nd^ edition BDI-II ([Beck et al., 1996](#_ENREF_3)) was selected to control for manifestations of depression; and the German version ([Margraf and Ehlers, 2007](#_ENREF_13)) of the Beck Anxiety Inventory BAI ([Beck and Steer, 1993](#_ENREF_2)) was chosen to evaluate anxiety symptoms in response to mTBI.

**Magnetic resonance imaging data acquisition**

MRI scans were acquired on a 3.0 Tesla Philips Ingenia whole body scanner (Philips Medical Systems, Best, The Netherlands) equipped with a transmit-receive body coil and a commercial 15-elements transmit-receive head coil array that is capable of sensitivity encoding (SENSE).

Resting-state functional MRI images were obtained using an echo-planar imaging sequence with an acquired spatial resolution of 3.0 x 3.0 x 3.0 mm^3^ (acquisition matrix: 72 x 74 pixel, 45 axial slices) and reconstructed to a spatial resolution of 1.72 x 1.72 x 3 mm^3^ (reconstruction matrix: 128 x 128 pixel, 45 axial slices). Further imaging parameters were: Field of view (FOV) = 220 x 220 mm^2^; slice thickness = 3 mm; number of slices = 45; brain volumes = 140; repetition time (TR) = 2,220 ms; echo time (TE) = 15.19 ms; acquisition time = 5.19 minutes; flip angle (α) = 78°and SENSE factor (R) = 1.8. During resting state data acquisition subjects were instructed to keep their eyes closed and to let their minds wandering.

A diffusion-weighted spin echo echo-planar imaging (EPI) sequence was used to obtain diffusion-weighted scans with a measured and reconstructed spatial resolution of 2.0 x 2.0 x 2.0 mm^3^ (acquisition and reconstruction matrix 112 x 112 pixels, 75 slices). Further imaging parameters were: FOV = 224 x 224 mm^2^; TE = 64.90 ms; repetition time = 18.714 s; α = 90°; SENSE factor R = 2.1; b-value b = 1,000 s/mm^2^; and number of averages = 1, acquisition time 23:05 minutes. Diffusion was measured along 64 non-collinear directions preceded by a non-diffusion-weighted volume (reference volume).

A T1-weighted fast field echo (FFE) sequence was used to map the B_0_ field in order to correct the DTI data for EPI-related geometrical distortions. The B_0_ map (3D echo sequence) is composed of a magnitude and a phase image and was measured with a spatial resolution of 2.0 x 2.0 x 4.0 mm^3^ (acquisition matrix 112 × 56 pixels, 75 slices) and reconstructed to a spatial resolution of 2.0 x 2.0 x 2.0 mm^3^ (acquisition matrix 112 × 112 pixels, 75 slices). Further imaging parameters were: FOV = 224 × 224 mm^2^, 75 slices, dual TE = 3.60 / 5.63 ms, TR = 30.0 ms, α = 60°, acquisition time (min) 4:11.

A volumetric 3D T1-weighted gradient echo sequence (turbo field echo) image was measured with a spatial resolution of 1.0 × 1.0 × 1.0 mm^3^ (acquisition matrix 240 × 240 pixels, 160 sagittal slices) and reconstructed to a spatial resolution of 0.94 × 0.94 × 1.0 mm^3^ (reconstruction matrix 256 × 256 pixels, 160 sagittal slices). Further imaging parameters were: FOV = 240 × 240 mm^2^, slice thickness = 1 mm, number of slices = 160, TE = 3.70 ms, TR = 8.14 ms, α = 8°, SENSE factor R = 1.8, acquisition time 7:29 minutes.

**Brain network construction**

We used the popular automated anatomical labelling (AAL) atlas (Tzourio-Mazoyer et al., 2002) in order to define the nodes of the functional and structural brain connectome. We used the AAL version with 90 cortical and subcortical anatomical regions (45 regions of interest for each hemisphere) excluding the cerebellum.

**Preprocessing of resting-state fMRI data and construction of the functional connectivity network**

Functional resting-state MRI data were preprocessed with Data Processing Assistant for Resting-State fMRI (DPARSFA) toolbox version 3.1 ([Chao-Gan and Yu-Feng, 2010](#_ENREF_8)) set within the Data Processing & Analysis of Brain Imaging (DPABI) toolbox of SPM8 (<http://www.fil.ion.ucl.ac.uk/spm/software/spm8/>).

Data preprocessing of functional connectivity included the following steps: 1) slice timing correction (mid-slice was used as reference slice); 2) realignment and extraction of head motion parameters according the approach proposed by Power and colleagues ([Power et al., 2012](#_ENREF_15); [Power et al., 2015](#_ENREF_16)); 3) nuisance covariates regression: Friston-24-parameter model. In addition, the global mean, white matter and the cerebrospinal fluid signal were also removed to reduce the effects of non-neuronal BOLD fluctuations; 4) linear- and non-linear spatial normalization to MNI space by using unified segmentation of the T1 image; 5) voxel re-sampling to 2 x 2 x 2 mm^3^; 6) smoothing with a 4 mm full-width-half-maximum (FWHM) Gaussian kernel; 7) removal of very low (< 0.01 Hz) and high frequencies band (> 0.1 Hz) reported to be of physiological importance ([Biswal et al., 1995](#_ENREF_5); [Cordes et al., 2001](#_ENREF_9)) and 8) time courses were obtained for all regions of interests and then correlated (Pearson) in a region-wise (AAL) manner to generate functional connectivity matrices. To improve the normality of the distribution, these connectivity maps were then converted into z-score maps by Fisher’s r-to-z-transformation ([Chao-Gan and Yu-Feng, 2010](#_ENREF_8)). Each cell in the connectivity matrix contained the correlation value of the preprocessed time series between the ith and the jth node ([Zalesky et al., 2010](#_ENREF_24)). Finally, these steps resulted in an undirected, weighted 90 x 90 nodes connectivity matrix for each subject.

**Preprocessing of DTI data and construction of the structural connectivity network**

Preprocessing of the diffusion-weighted MRI data was performed with FSL tools (FMRIB software library; version 5.0.6; http://www.fmrib.ox.ac.uk/fsl/) ([Smith et al., 2004](#_ENREF_18)) such as the FDT (FMRIB diffusion toolbox; version 3.0, default parameters) ([Behrens et al., 2003](#_ENREF_4)). For deterministic fibre tractography we used the Diffusion Toolkit (DTK, version 0.6.2.1) and TrackVis software (version 0.5.2.1; http://trackvis.org/) ([Park et al., 2009](#_ENREF_14)). The connectivity matrix was computed in MATLAB (version 8.0.0.783; <http://www.mathworks.com/index.html>). To construct the connectivity matrix of the WM pathways, the following fully automated preprocessing steps were realized: 1) In a first step, a binary brain mask was created using FSL’s brain extraction tool (BET). This mask is used in later steps to exclude non-brain tissue. 2) Eddy current distortions and head movements were corrected using the EDDY_CORRECT tool of FDT. In addition, we performed separate analysis using the tool “eddy” (without the supplementary combination “topup” since we did not acquire b0 images with reverse phase encoding directions) and included the average (after z-score transformations) of the mean of the translational and rotational motion estimation parameters (average volume-by-volume translations and average volume-by-volume rotations) as a nuisance regressor in the analysis with NBS. We included the average of the mean translational and rotational motion estimation parameters as only one nuisance regressor in order to preserve one degree of freedom 3) EPI-related geometrical distortions due to magnetic field inhomogeneities were unwarped using the B_0_ map and FSL’s FUGUE tool. 4) Diffusion gradients were adjusted for rotations introduced by the eddy current and head movement corrections. 5) The preprocessed DTI data were then subjected to the DTK to compute voxel-wise diffusion tensors and to construct the (principal) eigenvector and eigenvalue maps as well as a map of fractional anisotropy (FA). 6) Deterministic tractography was conducted in DTK using the “brute force” approach with an interpolated streamline tracking algorithm. Twenty streamlines per voxel were propagated and fibre tracking was stopped if FA was lower than 0.10 or if the turning angle of a streamline between two consecutive voxels was larger than 45°. This resulted in a whole brain connectome comprised by about 2-3 millions of streamlines including subcortical pathways and connections to the cerebellum. 7) The individual FA map was registered onto the FMRIB58-FA template, which is in correspondence with the MNI152 standard space, using FSL’s linear image registration tool (FLIRT) and the resulting transformations were stored. Due to the nature of the streamlines we run FLIRT and not FNIRT. Applying nonlinear transformations can result in „tearing“ streamlines (and in addition, longer streamlines are more prone to tear) so that when computing the connectivity measure (number of streamlines between two brain regions in our study) „teared“ streamlines are not counted any longer. 8) These transformations were then applied to the streamlines produced in step 6 in order to transform the streamlines into the MNI152 space. It is important to note that spatial normalization into standard space has been applied after tractography had been performed in native space, i.e. only the resulting streamlines (reconstructed white matter fibres) were transformed, not the preprocessed DTI images that are used for tractography. 9) The automated anatomical labeling (AAL) regions of interest (ROIs) ([Tzourio-Mazoyer et al., 2002](#_ENREF_20)), which are already in MNI152 standard space, were used to count the number of streamlines between each pair of ROIs. This AAL template consists of 90 ROIs (45 in each hemisphere) covering the entire neocortex (78 cortical ROIs) as well as the subcortical structures amygdala, hippocampus, thalamus, caudate, putamen, and pallidum (12 subcortical ROIs). 10) Streamlines connected to the cerebellum, those running through the brainstem, and streamlines shorter than 5 mm in length were removed (denoted streamlines omitted). Streamlines that make connections within a ROI itself were deleted (denoted selfloops). The number of the remaining streamlines between any pair of ROIs (denoted streamlines used to populate matrix) was counted using MATLAB scripts ([Zalesky et al., 2010](#_ENREF_24)). 11) This procedure resulted in an undirected, weighted 90 x 90 nodes (45 nodes per hemisphere) connectivity matrix for each individual participant. The strength of a structural connection was operationalized by the number of reconstructed streamlines between two ROIs. 12) The undirected, weighted 90 x 90 nodes connectivity matrices were then subjected to a network-based statistical analysis (NBS) ([Zalesky et al., 2010](#_ENREF_24)).

In the case of any structural subnetwork showing group differences or interactions between group and time in number of streamlines, mean fractional anisotropy (FA) values were calculated for each streamline and then also tested in network-based statistics to explore the linkage between the more traditional measure of FA and the network-based mapping measure used in the present study, i.e. the number of reconstructed streamlines.

**Supplementary Results**

**Acute and longitudinal alterations in fractional anisotropy**

The increase in number of streamlines in the mTBI group corresponded to a significant increase in FA in 35 out of the 53 investigated edges (Cohen’s d = -1.56, CI = -2.012– -1.108, p < 0.001, Supplementary Fig. 1 and Supplementary Table 3). Furthermore, increased FA in this 35-edge subnetwork was positively correlated with the number of streamlines within the 53-edge subnetwork (r = 0.419, p = 0.003). The group x time interaction analysis of FA yielded a subnetwork of 10 edges distributed over 11 nodes indicating a weak trend towards decreased mean FA for the patients at Visit 2 (Cohen’s d = -0.6, CI = -1.005– -0.195, p = 0.119, Supplementary Fig. 2 and Supplementary Table 5). Within the patients group, the longitudinal FA change of this subnetwork was furthermore positively correlated with the longitudinal alterations of the 19 edge-structural connectivity subnetwork, rather unexpectedly since they displayed a similar topographical pattern (r = 0.280, p = 0.026, one-sided).

**Comparison between correction methods for eddy current-induced distortions**

The whole-brain group comparison analysis in the acute phase using “eddy” together with head motion correction yielded qualitatively comparable results as those obtained when using eddy_correct (Supplementary Fig. 3 and Supplementary Fig. 4). Selecting approximately the same set t-threshold of 1.8 the size of the obtained subnetwork (encompassing now 51 nodes) and the direction of the group difference (showing again an increase in structural connectivity in patients compared to healthy controls) were conserved. Common nodes between the two subnetworks were 41, 22 in the left and 19 in the right hemisphere.

**Acute alterations in global efficiency and normalized characteristic path length**

Aspects of segregation (clustering, modularity), integration (characteristic path length, global efficiency) and centrality (degree, betweenness centrality) are clearly different from strength of connectivity calculated by using the number of reconstructed streamlines between any two nodes. It seems intuitive that a direct comparison is not feasible. Nevertheless, to dissolve the apparent contradiction, we run new analyses examining the entire connectivity matrix at Visit 1, but adopting the graph theoretical measure “global efficiency” (average inverse shortest path length) and “normalized characteristic path length” (characteristic path length of the network normalized to an appropriate null network). Consistently with the literature, there was a decrease in global efficiency and an increase in normalized characteristic path length in mTBI patients compared with healthy controls ([Caeyenberghs et al., 2012](#_ENREF_6); [Caeyenberghs et al., 2014](#_ENREF_7); [Yuan et al., 2014](#_ENREF_23)). Both network-level measures of structural connectivity did not however reach a statistical significance (p > 0.409). This was probably due to the fact that we explored group-wise differences at large-scale instead of within “our 53-edge structural subnetwork” that demonstrated increased connectivity in the acute phase.

**2 Supplementary Figures and Tables**

**2.1 Supplementary Tables**

**Supplementary Table 1: Neuropsychological assessment scores (uncorrected and adjusted p-values for multiple comparisons using false discovery rate)**

|  | **Visit 1 (acute phase) Group differences (unpaired t-test)** | | **Visit 2 (chronic phase) Group differences (unpaired t-test)** | | **Changes within groups over time (paired t-test)** | | | |
| --- | --- | --- | --- | --- | --- | --- | --- | --- |
|  | **p-value (uncorrected)** | **p-value (adjusted for FDR)** | **p-value (uncorrected)** | **p-value (adjusted for FDR)** | **Patients  (uncorrected p-value)** | **Patients  (adjusted for FDR)** | **Controls (uncorrected p-value)** | **Controls (adjusted for FDR)** |
| **Neuropsychological assessment** |  |  |  |  |  |  |  |  |
| RPQ (total score) | <0.001 | 0.006 | 0.005 | 0.019 | <0.001 | 0.006 | 0.537 | 0.612 |
| Alertness, tonic (ms) | 0.007 | 0.021 | 0.593 | 0.660 | 0.004 | 0.018 | 0.156 | 0.232 |
| Alertness, phasic (ms) | 0.031 | 0.065 | 0.992 | 0.992 | 0.005 | 0.019 | 0.098 | 0.160 |
| Go/Nogo (ms) | 0.019 | 0.047 | 0.059 | 0.100 | 0.019 | 0.047 | 0.042 | 0.081 |
| Go/Nogo (errors) | 0.606 | 0.660 | 0.828 | 0.845 | 0.726 | 0.757 | 0.453 | 0.555 |
| Divided attention, auditory (ms) | 0.145 | 0.222 | 0.134 | 0.212 | 0.025 | 0.058 | 0.013 | 0.038 |
| Divided attention, visual (ms) | 0.014 | 0.038 | 0.046 | 0.084 | <0.001 | 0.006 | <0.001 | 0.006 |
| Working memory | 0.050 | 0.088 | 0.641 | 0.683 | 0.001 | 0.006 | 0.391 | 0.498 |
| AVLGT recall score | 0.178 | 0.249 | 0.326 | 0.432 | <0.001 | 0.006 | <0.001 | 0.006 |
| AVLGT long delay | 0.476 | 0.560 | 0.480 | 0.560 | 0.002 | 0.011 | <0.001 | 0.006 |
| BDI-II (score) | 0.006 | 0.021 | 0.167 | 0.241 | 0.003 | 0.015 | 0.275 | 0.374 |
| BAI (score) | 0.032 | 0.065 | 0.007 | 0.021 | 0.396 | 0.498 | 0.043 | 0.081 |
| Intellectual ability (IQ) | 0.031 | 0.065 | - | - | - | - | - | - |
| AVLGT = German adaptation of the Rey Auditory Verbal Learning Tests RAVLT; BAI = Beck Anxiety Inventar; BDI-II = Beck Depression Inventory, 2nd edition; FDR = false discovery rate; ms = ms milliseconds; RPQ = Rivermead Post-Concussion Symptoms Questionnaire. The adjustment for false discovery rate occurred over all 49 tests (<https://brainder.org/2011/09/05/fdr-corrected-fdr-adjusted-p-values/>). | | | | | | | | |

| **Supplementary Table 2: Reduced resting-state functional connectivity in a 15-edge subnetwork for patients compared to controls at Visit 1.** | | | | | |
| --- | --- | --- | --- | --- | --- |
| List of functional connections | | | | |  |
|  |  |  | t-value |  |  |
| Precuneus_L |  | Heschl_L | 3.87 |  |  |
| Heschl_R |  | Temporal_Pole_Sup_L | 3.81 |  |  |
| Cingulum_Ant_R |  | ParaHippocampal_R | 3.77 |  |  |
| Cingulum_Ant_L |  | ParaHippocampal_R | 3.61 |  |  |
| Cingulum_Ant_L |  | Cingulum_Post_R | 3.60 |  |  |
| Cingulum_Ant_L |  | Cingulum_Post_L | 3.52 |  |  |
| Precuneus_L |  | Heschl_R | 3.49 |  |  |
| Cingulum_Post_L |  | Temporal_Sup_R | 3.41 |  |  |
| Precuneus_R |  | Heschl_L | 3.36 |  |  |
| Amygdala_R |  | Heschl_R | 3.36 |  |  |
| Temporal_Sup_L |  | Temporal_Pole_Mid_R | 3.31 |  |  |
| Cingulum_Ant_R |  | Cingulum_Post_L | 3.30 |  |  |
| Supp_Motor_Area_R |  | Cingulum_Ant_L | 3.23 |  |  |
| Heschl_R |  | Temporal_Pole_Mid_R | 3.23 |  |  |
| ParaHippocampal_R |  | Heschl_L | 3.17 |  |  |

**Legend:** Cohen’s d = 1.59, CI = 1.138–2.046, p = 0.0057.

Abbreviations: Ant, anterior; L, left; Mid, middle; Post, posterior; R, right; Sup, superior; Supp, supplementary.

| **Supplementary Table 3: Increased DTI-based structural connectivity in a**  **53-edge subnetwork for patients compared to controls at Visit 1.** | | | | | | | | | | | |
| --- | --- | --- | --- | --- | --- | --- | --- | --- | --- | --- | --- |
| List of structural connections | | | | | | | | |  |  |  |
|  |  | |  | | t-value (streamlines) | | t-value  (FA) | |  |  |  |
| Fusiform_L | |  | | Temporal_Inf_L | | 3.46 |  | 0.99 | | |  |
| Rolandic_Oper_L | |  | | Heschl_L | | 2.87 |  |  | |  | |
| Cingulum_Post_R | |  | | Lingual_R | | 2.78 |  |  | |  | |
| Frontal_Sup_Medial_L | |  | | Cingulum_Ant_L | | 2.76 |  | 1.35 | |  | |
| Olfactory_R | |  | | ParaHippocampal_R | | 2.74 |  | 1.16 | |  | |
| Rectus_L | |  | | Pallidum_L | | 2.48 |  | 0.02 | |  | |
| Insula_R | |  | | Temporal_Pole_Sup_R | | 2.48 |  |  | |  | |
| Precentral_L | |  | | Thalamus_L | | 2.46 |  | 1.41 | |  | |
| Supp_Motor_Area_R | |  | | Frontal_Sup_Medial_R | | 2.43 |  | 0.94 | |  | |
| Fusiform_R | |  | | Temporal_Mid_R | | 2.41 |  | 0.19 | |  | |
| Caudate_L | |  | | Putamen_R | | 2.32 |  | 0.15 | |  | |
| ParaHippocampal_R | |  | | Temporal_Mid_R | | 2.3 |  | 2.33 | |  | |
| Insula_L | |  | | Temporal_Pole_Mid_L | | 2.28 |  | 3.17 | |  | |
| Putamen_L | |  | | Putamen_R | | 2.28 |  | 1.15 | |  | |
| Precuneus_R | |  | | Thalamus_R | | 2.27 |  | 1.90 | |  | |
| Calcarine_L | |  | | Precuneus_R | | 2.23 |  | 0.63 | |  | |
| Frontal_Mid_R | |  | | Putamen_R | | 2.23 |  |  | |  | |
| Frontal_Sup_Medial_R | |  | | Putamen_R | | 2.21 |  | 0.88 | |  | |
| Occipital_Mid_L | |  | | Temporal_Mid_L | | 2.2 |  | 0.29 | |  | |
| Putamen_L | |  | | Pallidum_L | | 2.19 |  | 0.03 | |  | |
| Postcentral_L | |  | | Hippocampus_R | | 2.19 |  | 2.81 | |  | |
| Postcentral_L | |  | | Putamen_L | | 2.18 |  | 3.21 | |  | |
| Supp_Motor_Area_L | |  | | Thalamus_L | | 2.17 |  | 1.76 | |  | |
| Supp_Motor_Area_L | |  | | Precuneus_L | | 2.16 |  | 1.71 | |  | |
| Cingulum_Post_R | |  | | Fusiform_R | | 2.16 |  | 2.38 | |  | |
| Amygdala_R | |  | | Thalamus_R | | 2.16 |  | 1.85 | |  | |
| Insula_R | |  | | ParaHippocampal_R | | 2.14 |  |  | |  | |
| Frontal_Sup_Orb_R | |  | | Cingulum_Ant_R | | 2.11 |  |  | |  | |
| Cingulum_Post_L | |  | | Precuneus_L | | 2.1 |  |  | |  | |
| Calcarine_R | |  | | Lingual_R | | 2.1 |  |  | |  | |
| Putamen_L | |  | | Frontal_Med_Orb_R | | 2.03 |  | 2.72 | |  | |
| Frontal_Sup_R | |  | | Cingulum_Post_R | | 2.02 |  |  | |  | |
| Parietal_Sup_R | |  | | Temporal_Mid_R | | 2.02 |  | 0.33 | |  | |
| Frontal_Mid_Orb_L | |  | | Supp_Motor_Area_L | | 2.01 |  | 1.19 | |  | |
| Frontal_Mid_Orb_R | |  | | Cingulum_Ant_R | | 2.01 |  |  | |  | |
| Supp_Motor_Area_L | |  | | Olfactory_L | | 1.99 |  | 2.86 | |  | |
| Thalamus_L | |  | | Temporal_Mid_L | | 1.99 |  | 1.31 | |  | |
| Insula_R | |  | | Temporal_Sup_R | | 1.98 |  |  | |  | |
| Frontal_Sup_R | |  | | Frontal_Sup_Orb_R | | 1.97 |  |  | |  | |
| Olfactory_R | |  | | Caudate_R | | 1.97 |  |  | |  | |
| Fusiform_R | |  | | Precuneus_R | | 1.96 |  | 2.08 | |  | |
| Frontal_Sup_Orb_L | |  | | Putamen_L | | 1.95 |  | 0.01 | |  | |
| Rolandic_Oper_L | |  | | Temporal_Pole_Sup_L | | 1.95 |  |  | |  | |
| Rectus_L | |  | | Insula_L | | 1.94 |  | 0.95 | |  | |
| Fusiform_L | |  | | Temporal_Mid_L | | 1.93 |  | 2.25 | |  | |
| Frontal_Sup_Orb_L | |  | | Cingulum_Ant_L | | 1.92 |  |  | |  | |
| Supp_Motor_Area_L | |  | | Amygdala_L | | 1.92 |  | 0.79 | |  | |
| Occipital_Mid_R | |  | | Temporal_Mid_R | | 1.91 |  |  | |  | |
| Precentral_L | |  | | Cingulum_Ant_L | | 1.88 |  | 1.71 | |  | |
| Amygdala_L | |  | | Pallidum_L | | 1.88 |  | 0.79 | |  | |
| Parietal_Sup_R | |  | | Temporal_Sup_R | | 1.88 |  |  | |  | |
| Hippocampus_R | |  | | Temporal_Mid_R | | 1.88 |  | 0.73 | |  | |
| Insula_L | |  | | Temporal_Pole_Sup_L | | 1.87 |  |  | |  | |

**Legend:**

Analysis with number of streamlines: Cohen’s d = -1.71, CI = -2.168– -1.243,

p = 0.041.

Analysis with fractional anisotropy-value: Cohen’s d = -1.56, CI = -2.012– -1.108,

p < 0.001.

Abbreviations: Ant, anterior; FA, fractional anisotropy; Inf, inferior; L, left; Med, medialis; Mid, middle; Oper, operculum; Orb, orbitalis; Post, posterior; R, right; Sup, superior; Supp, supplementary.

**Supplementary Table 4: Functional connectivity: selective interaction within the 15-edge subnetwork of interest resulting from group comparison at Visit 1.**

| List of functional connections | | | | | | | | |
| --- | --- | --- | --- | --- | --- | --- | --- | --- |
|  |  | | |  |  | | t-value | |
| Cingulum_Post_L | |  | Temporal_Sup_R | | | 3.33 | |  |
| Temporal_Sup_L | |  | Temporal_Pole_Mid_R | | | 3.02 | |  |
| Cingulum_Ant_L | |  | ParaHippocampal_R | | | 2.33 | |  |
| Heschl_R | |  | Temporal_Pole_Mid_R | | | 2.23 | |  |
| Cingulum_Ant_R | |  | ParaHippocampal_R | | | 2.17 | |  |
| Amygdala_R | |  | Heschl_R | | | 2.08 | |  |
| Heschl_R | |  | Temporal_Pole_Sup_L | | | 1.96 | |  |
| Cingulum_Ant_L | |  | Cingulum_Post_L | | | 1.95 | |  |
| Cingulum_Ant_L | |  | Cingulum_Post_R | | | 1.82 | |  |
| Cingulum_Ant_R | |  | Cingulum_Post_L | | | 1.51 | |  |
| ParaHippocampal_R | |  | Heschl_L | | | 1.1 | |  |
| Precuneus_L | |  | Heschl_L | | | 1.09 | |  |
| Precuneus_L | |  | Heschl_R | | | 1.08 | |  |
| Precuneus_R | |  | Heschl_L | | | 0.99 | |  |
| Supp_Motor_Area_R | |  | Cingulum_Ant_L | | | 0.93 | |  |

**Legend:** Cohen’s d = 0.9, CI = 0.490–1.321, p = 0.002.

Abbreviations: Ant, anterior; L, left; Mid, middle; Post, posterior; R, right; Sup, superior; Supp, supplementary.

**Supplementary Table 5: Structural connectivity: selective interaction within the**

**53-edge subnetwork (streamlines) respectively the 35-edge subnetwork (fractional anisotropy values) of interest resulting from group comparison at Visit 1.**

| List of structural connections | | | | | | | | |  |  |
| --- | --- | --- | --- | --- | --- | --- | --- | --- | --- | --- |
| Network | | |  | |  | t-value t-value (FA)  (streamlines) | | |  |  |
| 1 |  | |  |  | | |  | |  |  |
|  | Precentral_L | |  | Cingulum_Ant_L | | 2.21 | | 0.92 |  |  |
|  | Putamen_L | |  | Frontal_Med_Orb_R | | 2.16 | |  |  |  |
|  | Frontal_Sup_Orb_L | |  | Putamen_L | | 2.07 | |  |  |  |
|  | Supp_Motor_Area_R | |  | Frontal_Sup_Medial_R | | 1.69 | |  |  |  |
|  | Frontal_Sup_Medial_L | |  | Cingulum_Ant_L | | 1.44 | | 0.1 |  |  |
|  | Putamen_L | |  | Pallidum_L | | 1.24 | |  |  |  |
|  | Caudate_L | |  | Putamen_R | | 1.19 | |  |  |  |
|  | Supp_Motor_Area_L | |  | Amygdala_L | | 1.06 | |  |  |  |
|  | Putamen_L | |  | Putamen_R | | 0.77 | |  |  |  |
|  | Frontal_Mid_Orb_L | |  | Supp_Motor_Area_L | | 0.69 | | 1.88 |  |  |
|  | Postcentral_L | |  | Putamen_L | | 0.62 | |  |  |  |
|  | Precentral_L | |  | Thalamus_L | | 0.55 | | 0.13 |  |  |
|  | Frontal_Sup_Orb_L | |  | Cingulum_Ant_L | | 0.5 | |  |  |  |
|  | Supp_Motor_Area_L | |  | Olfactory_L | | 0.41 | | 0.17 |  |  |
|  | Supp_Motor_Area_L | |  | Thalamus_L | | 0.32 | | 1.3 |  |  |
|  | Amygdala_L | |  | Pallidum_L | | 0.22 | |  |  |  |
|  | Supp_Motor_Area_L | |  | Precuneus_L | | 0.19 | | 0.15 |  |  |
|  | Frontal_Mid_R | |  | Putamen_R | | 0.14 | |  |  |  |
|  | Frontal_Sup_Medial_R | |  | Putamen_R | | 0.03 | |  |  |  |
|  |  | |  |  | |  | |  |  |  |
| 2 |  |  |  |  |  |  |  |  |  |  |
|  | Calcarine_L | |  | Precuneus_R | | 2.19 | |  |  |  |
|  | Frontal_Sup_Orb_R | |  | Cingulum_Ant_R | | 2.04 | |  |  |  |
|  | Amygdala_R | |  | Thalamus_R | | 1.93 | |  |  |  |
|  | Hippocampus_R | |  | Temporal_Mid_R | | 1.85 | |  |  |  |
|  | Cingulum_Post_R | |  | Fusiform_R | | 1.83 | |  |  |  |
|  | Precuneus_R | |  | Thalamus_R | | 1.77 | |  |  |  |
|  | ParaHippocampal_R | |  | Temporal_Mid_R | | 1.75 | |  |  |  |
|  | Parietal_Sup_R | |  | Temporal_Sup_R | | 1.7 | |  |  |  |
|  | Frontal_Sup_R | |  | Frontal_Sup_Orb_R | | 1.67 | |  |  |  |
|  | Occipital_Mid_R | |  | Temporal_Mid_R | | 1.52 | |  |  |  |
|  | Fusiform_R | |  | Precuneus_R | | 1.43 | |  |  |  |
|  | Frontal_Sup_R | |  | Cingulum_Post_R | | 1.25 | |  |  |  |
|  | Olfactory_R | |  | ParaHippocampal_R | | 1.2 | |  |  |  |
|  | Parietal_Sup_R | |  | Temporal_Mid_R | | 0.9 | |  |  |  |
|  | Frontal_Mid_Orb_R | |  | Cingulum_Ant_R | | 0.89 | |  |  |  |
|  | Cingulum_Post_R | |  | Lingual_R | | 0.66 | |  |  |  |
|  | Calcarine_R | |  | Lingual_R | | 0.57 | |  |  |  |
|  | Fusiform_R | |  | Temporal_Mid_R | | 0.34 | |  |  |  |

**Legend:**

Analysis with number of streamlines: Subnetwork 1: Cohen’s d = -0.72, CI = -1.132– -0.315, p = 0.025. Subnetwork 2: Cohen’s d = -0.71, CI = -1.120– -0.303, p = 0.035.

Analysis with fractional anisotropy-value: Cohen’s d = -0.6, CI = -1.005– -0.195, p = 0.119. In addition to the edges listed above, the interaction analysis revealed also three more connections: Fusiform_L to Temporal_Mid_L = 2.19, Thalamus_L to Temporal_Mid_L = 0.44, Fusiform_L to Temporal_Inf_L = 0.18.

Abbreviations: Ant, anterior; FA, fractional anisotropy; Inf, inferior; L, left; Med, medialis; Mid, middle; Orb, orbitalis; Post, posterior; R, right; Sup, superior; Supp, supplementary.

**Supplementary Table 6: Functional connectivity: whole-brain group x time interaction in subnetwork of 59 edges and 48 nodes**

| List of functional connections | | | | | | |  |
| --- | --- | --- | --- | --- | --- | --- | --- |
|  |  | |  | | t-value | |  |
| Occipital_Mid_R |  | | Temporal_Pole_Sup_L | | 4.01 |  |  |
| Frontal_Med_Orb_L | |  | Cingulum_Mid_L | 3.91 | |  |  |
| Paracentral_Lobule_L | |  | Thalamus_L | 3.53 | |  |  |
| Frontal_Med_Orb_R | |  | Cingulum_Mid_L | 3.5 | |  |  |
| Frontal_Sup_Medial_R | |  | Temporal_Sup_R | 3.48 | |  |  |
| Frontal_Sup_Orb_L | |  | Angular_R | 3.35 | |  |  |
| Occipital_Sup_R | |  | Temporal_Pole_Sup_L | 3.35 | |  |  |
| Cingulum_Post_L | |  | Temporal_Sup_R | 3.33 | |  |  |
| Cuneus_L | |  | Temporal_Pole_Sup_L | 3.3 | |  |  |
| Olfactory_R | |  | Calcarine_L | 3.14 | |  |  |
| Cuneus_R | |  | Temporal_Pole_Sup_L | 3.14 | |  |  |
| Occipital_Sup_R | |  | Occipital_Mid_R | 3.1 | |  |  |
| Frontal_Med_Orb_R | |  | Putamen_L | 3.08 | |  |  |
| Cingulum_Post_L | |  | Caudate_R | 3.03 | |  |  |
| Occipital_Mid_R | |  | Temporal_Pole_Mid_L | 3.02 | |  |  |
| Temporal_Sup_L | |  | Temporal_Pole_Mid_R | 3.02 | |  |  |
| Frontal_Mid_Orb_R | |  | Frontal_Sup_Medial_R | 2.97 | |  |  |
| Frontal_Med_Orb_R | |  | Cingulum_Mid_R | 2.97 | |  |  |
| Frontal_Inf_Orb_L | |  | Cuneus_L | 2.95 | |  |  |
| Thalamus_R | |  | Temporal_Mid_L | 2.92 | |  |  |
| Frontal_Med_Orb_L | |  | Temporal_Sup_R | 2.89 | |  |  |
| Frontal_Inf_Orb_L | |  | Occipital_Mid_R | 2.85 | |  |  |
| Olfactory_L | |  | Cingulum_Mid_R | 2.78 | |  |  |
| Frontal_Med_Orb_R | |  | Putamen_R | 2.77 | |  |  |
| Cuneus_R | |  | Thalamus_L | 2.77 | |  |  |
| Frontal_Med_Orb_L | |  | SupraMarginal_R | 2.76 | |  |  |
| Frontal_Med_Orb_L | |  | Putamen_L | 2.76 | |  |  |
| Cingulum_Post_L | |  | Temporal_Pole_Sup_R | 2.76 | |  |  |
| ParaHippocampal_L | |  | Cuneus_L | 2.74 | |  |  |
| Thalamus_R | |  | Temporal_Sup_L | 2.73 | |  |  |
| Frontal_Med_Orb_R | |  | Pallidum_R | 2.71 | |  |  |
| Hippocampus_L | |  | Occipital_Sup_R | 2.7 | |  |  |
| Frontal_Sup_Medial_L | |  | Temporal_Sup_R | 2.69 | |  |  |
| Frontal_Inf_Orb_L | |  | Cuneus_R | 2.66 | |  |  |
| Insula_L | |  | Temporal_Pole_Mid_R | 2.62 | |  |  |
| Angular_R | |  | Temporal_Pole_Sup_L | 2.59 | |  |  |
| Olfactory_L | |  | Calcarine_L | 2.58 | |  |  |
| Precuneus_L | |  | Thalamus_L | 2.58 | |  |  |
| Insula_R | |  | Temporal_Pole_Mid_R | 2.58 | |  |  |
| Frontal_Med_Orb_R | |  | Temporal_Sup_R | 2.57 | |  |  |
| Frontal_Inf_Oper_R | |  | Olfactory_L | 2.55 | |  |  |
| Supp_Motor_Area_L | |  | Heschl_R | 2.54 | |  |  |
| Cuneus_L | |  | Temporal_Pole_Sup_R | 2.53 | |  |  |
| Putamen_L | |  | Pallidum_L | 2.52 | |  |  |
| Rectus_R | |  | Occipital_Mid_R | 2.51 | |  |  |
| Frontal_Mid_Orb_L | |  | Angular_R | 2.51 | |  |  |
| Frontal_Sup_Medial_R | |  | Heschl_R | 2.51 | |  |  |
| Frontal_Med_Orb_L | |  | Cingulum_Mid_R | 2.5 | |  |  |
| ParaHippocampal_L | |  | Occipital_Sup_L | 2.5 | |  |  |
| Frontal_Sup_Orb_L | |  | Occipital_Mid_R | 2.46 | |  |  |
| Paracentral_Lobule_L | |  | Temporal_Sup_L | 2.46 | |  |  |
| Frontal_Med_Orb_L | |  | Pallidum_R | 2.45 | |  |  |
| Frontal_Inf_Oper_L | |  | Angular_R | 2.44 | |  |  |
| Cingulum_Mid_L | |  | Temporal_Pole_Sup_L | 2.44 | |  |  |
| Occipital_Sup_L | |  | Temporal_Pole_Sup_L | 2.44 | |  |  |
| Rolandic_Oper_L | |  | Paracentral_Lobule_L | 2.43 | |  |  |
| ParaHippocampal_R | |  | Pallidum_L | 2.43 | |  |  |
| Cuneus_L | |  | Thalamus_L | 2.43 | |  |  |
| Occipital_Sup_R | |  | Temporal_Pole_Mid_R | 2.42 | |  |  |
|  | |  |  |  | | | |

**Legend:** Cohen’s d = 1.87, CI = 1.402–2.353, p = 0.045.

Abbreviations: Inf, inferior; L, left; Med, medialis; Mid, middle;

Oper, operculum; Orb, orbitalis; Post, posterior; R, right;

Sup, superior; Supp, supplementary.

**Supplementary Table 7: Spearman rank-order correlations between changes in mean connectivity (functional and structural) and in cognitive performance across time points**

|  | **mTBI patients (*n* = 49)** | | |
| --- | --- | --- | --- |
|  | **Functional subnetwork (15 edges)** | **Structural**  **subnetwork 1**  **(19 edges)** | **Structural subnetwork 2**  **(18 edges)** |
| Alertness, tonic (RT) | rho = 0.044 p = 0.385 | rho = 0.098 p = 0.257 | rho = -0.070 p = 0.323 |
| Alertness, phasic (RT) | rho = 0.036 p = 0.405 | rho = 0.092 p = 0.271 | rho = 0.075 p = 0.310 |
| Go/Nogo (RT) | rho = -0.171 p = 0.128 | rho = 0.010 p = 0.473 | rho = -0.114 p = 0.225 |
| Divided attention auditory (RT) | rho = 0.053 p = 0.364 | rho = -0.083 p = 0.292 | **rho = -0.262** **p uncorrected = 0.039**  **p** **corrected = 0.264** |
| Divided attention visual (RT) | **rho = 0.333 p uncorrected = 0.012 p corrected = 0.120** | rho = 0.089 p = 0.278 | rho = -0.038 p = 0.401 |
| Working memory | **rho = -0.350 p uncorrected = 0.008 p corrected = 0.120** | rho = -0.168 p = 0.133 | rho = -0.101 p = 0.252 |
| Recall score (AVLGT) | rho = -0.044 p = 0.385 | **rho = 0.348 p uncorrected = 0.009**  **p corrected = 0.120** | rho = -0.194 p = 0.098 |
| Long delay (AVLGT) | rho = -0.183 p = 0.112 | rho = 0.091 p = 0.273 | rho = -0.126 p = 0.201 |
| BDI-II | rho = 0.108 p = 0.236 | **rho = 0.254** **p uncorrected = 0.0445**  **p corrected = 0.264** | rho = 0.019 p = 0.449 |
| BAI | rho = 0.054 p = 0.360 | rho = -0.097 p = 0.260 | rho = 0.111 p = 0.231 |
| AVLGT = German adaptation of the Rey Auditory Verbal Learning Tests RAVLT; BAI = Beck Anxiety Inventar; BDI-II = Beck Depression Inventory, 2nd edition; RT = reaction time. The adjustment for false discovery rate (FDR) occurred over all 49 tests (<https://brainder.org/2011/09/05/fdr-corrected-fdr-adjusted-p-values/>). P-values are reported one-tailed. Rho = partial Spearman's rank-order correlation. | | | |

**2.2 Supplementary Figures**


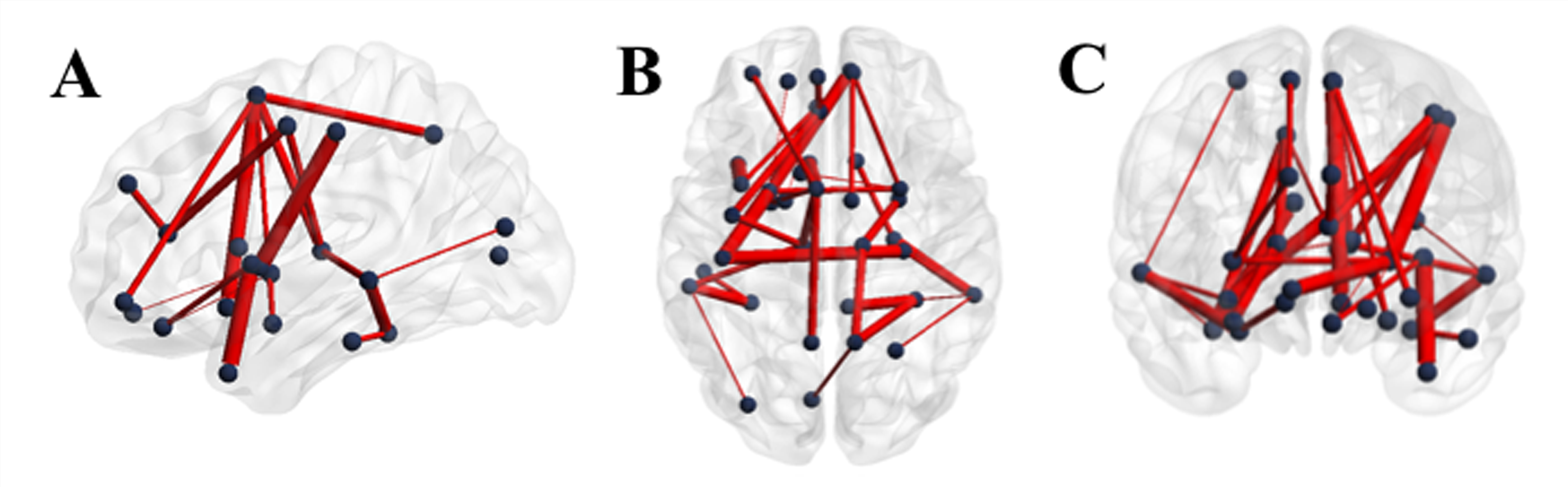


**Supplementary Figure 1** Increased fractional anisotropy in a 35-edge subnetwork deriving from the 53-edge subnetwork for patients compared to controls at Visit 1.

A = left, B = top, C = frontal. Cohen’s d = -1.56, CI = -2.012– -1.108, p < 0.001.

The NBS-specific set threshold was set to t = 0 in order to admit all possible connections of the 53-edge subnetwork to the set of suprathreshold links showing a change over time

**
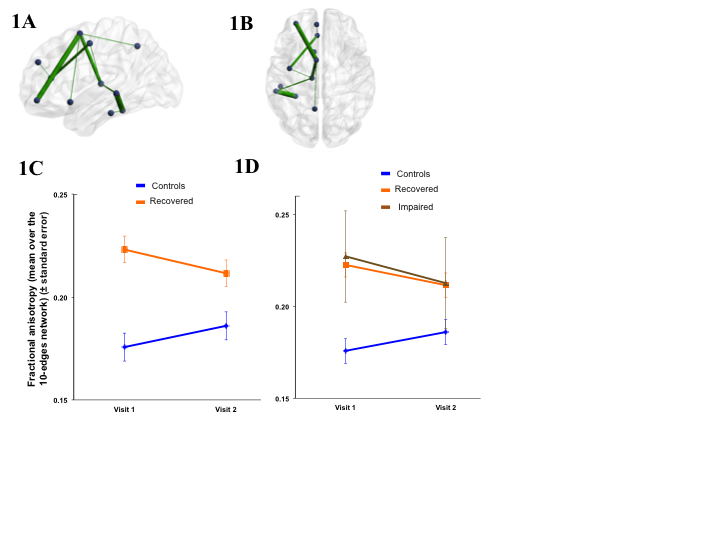
**

**Supplementary Figure 2** Changes in fractional anisotropy within the initially impaired 35-edge subnetwork over 1 year resulted in a 10-edge subnetwork (selective group x time interaction). Cohen’s d = -0.6, CI = -1.005– -0.195, p = 0.119. 1A= left, 1B = top, 1C = A decrease in FA in the patient sample and an increase in the control sample were responsible for the significant repeated-measures effect. The group effect at Visit 1 reached a strong significance (p < 0.001) that shifted into a weaker significance at Visit 2 (p = 0.004). The time effect for each group revealed trend-wise changes (patients p = 0.098, controls p = 0.152) over 1 year. 1D = At a descriptive level, the recovery curves of the subcohorts of patients with and without PCS were similar. The NBS-specific sensitivity threshold was set to t = 0 in order to admit all possible connections of the 35-edge subnetwork to the set of suprathreshold links showing a change over time

*
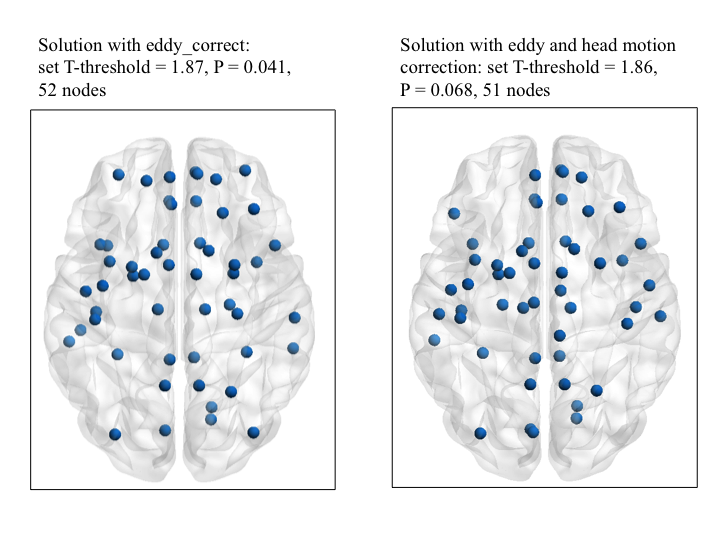
*

**Supplementary Figure 3** Comparison between correction efficiency of FSL tools: “eddy_correct” (left) against the combination of “eddy” with head motion estimation as a nuisance regressor at node level.

***
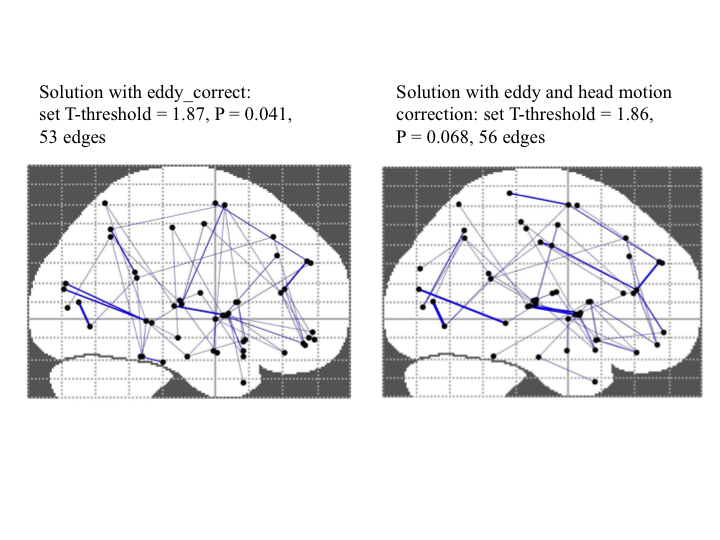
***

**Supplementary Figure 4** Comparison between correction efficiency of FSL tools: “eddy_correct” (left) against the combination of “eddy” with head motion estimation as a nuisance regressor at edge level.

**
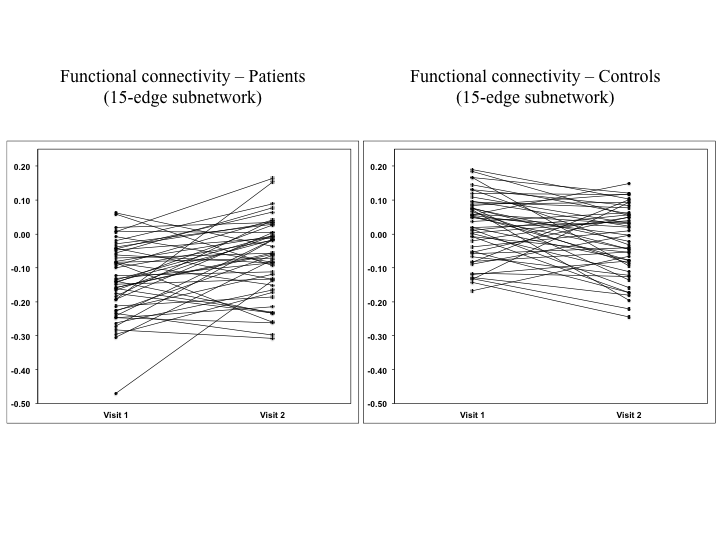
**

**Supplementary Figure 5** Functional connectivity changes (derived from the selective interaction analysis, see Figure 3 in the main manuscript) between visits of the 15-edge subnetwork separately for each patient (left) and each control (right).

**
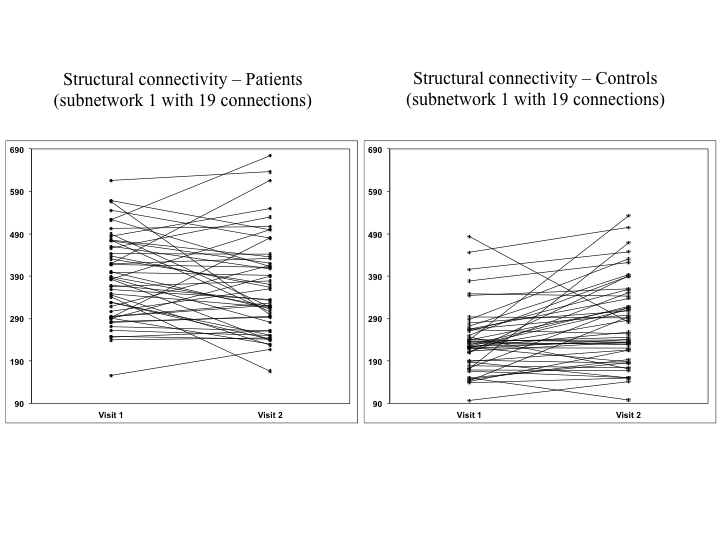
**

**Supplementary Figure 6** Structural connectivity changes (derived from the selective interaction analysis, see Figure 4 panels 1a-c in the main manuscript) between visits of the 19-edge left-hemispheric lateralized subnetwork separately for each patient (left) and each control (right).

**
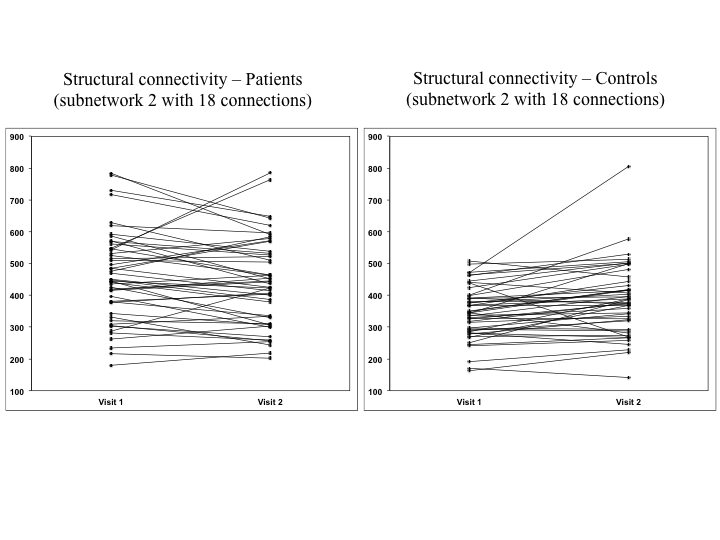
**

**Supplementary Figure 7** Structural connectivity changes (derived from the selective interaction analysis, see Figure 4 panels 2a-c in the main manuscript) between visits of the 18-edge right-hemispheric lateralized subnetwork separately for each patient (left) and each control (right).

**REFERENCES**

Balzer, C., Berger, J.-M., Caprez, G., Gonser, A., Gutbrod, K., and Keller, M. (2011). *Materialien und Normwerte für die neuropsychologische Diagnostik MNND. Testhandbuch.* Rheinfelden: Verlag Normdaten.

Beck, A.T., and Steer, R.A. (1993). *Beck Anxiety Inventory - Manual.* San Antonio, TX.: The Psychological Corporation.

Beck, A.T., Steer, R.A., and Brown, G.K. (1996). *Beck Depression Inventory - Second Edition. Manual.* San Antonio, TX.: The Psychological Corporation.

Behrens, T.E., Woolrich, M.W., Jenkinson, M., Johansen-Berg, H., Nunes, R.G., Clare, S., Matthews, P.M., Brady, J.M., and Smith, S.M. (2003). Characterization and propagation of uncertainty in diffusion-weighted MR imaging. *Magn Reson Med* 50**,** 1077-1088.

Biswal, B., Yetkin, F.Z., Haughton, V.M., and Hyde, J.S. (1995). Functional connectivity in the motor cortex of resting human brain using echo-planar MRI. *Magn Reson Med* 34**,** 537-541.

Caeyenberghs, K., Leemans, A., De Decker, C., Heitger, M., Drijkoningen, D., Linden, C.V., Sunaert, S., and Swinnen, S.P. (2012). Brain connectivity and postural control in young traumatic brain injury patients: A diffusion MRI based network analysis. *Neuroimage Clin* 1**,** 106-115.

Caeyenberghs, K., Leemans, A., Leunissen, I., Gooijers, J., Michiels, K., Sunaert, S., and Swinnen, S.P. (2014). Altered structural networks and executive deficits in traumatic brain injury patients. *Brain Struct Funct* 219**,** 193-209.

Chao-Gan, Y., and Yu-Feng, Z. (2010). DPARSF: A MATLAB Toolbox for "Pipeline" Data Analysis of Resting-State fMRI. *Front Syst Neurosci* 4**,** 13.

Cordes, D., Haughton, V.M., Arfanakis, K., Carew, J.D., Turski, P.A., Moritz, C.H., Quigley, M.A., and Meyerand, M.E. (2001). Frequencies contributing to functional connectivity in the cerebral cortex in "resting-state" data. *AJNR Am J Neuroradiol* 22**,** 1326-1333.

Formann, A.K., Waldherr, K., and Piswanger, K. (2011). *WMT-2. Wiener Matrizen-Test 2. Ein Rasch-skalierter spreachfreier Kurztest zur Erfassung der Intelligenz. Manual.* Göttingen: Beltz Test Gesellschaft.

Green, P. (2004). *Green’s Medical Symptom Validity Test (MSVT) for MicrosoftWindows. User’s manual.* Edmonton, Canada: Green’s Publishing Inc.

Hautzinger, M., Keller, F., and Kühner, C. (2006). *BDI-II. Beck-Depressions-Inventar Revision - Manual.* Frankfurt am Main: Harcourt Test Services GmbH.

Margraf, J., and Ehlers, A. (2007). *BAI. Beck Angst-Inventar - Manual. Deutsche Bearbeitung* Frankfurt am Main: Harcourt Test Services GmbH.

Park, J.H., Park, S.W., Kang, S.H., Nam, T.K., Min, B.K., and Hwang, S.N. (2009). Detection of traumatic cerebral microbleeds by susceptibility-weighted image of MRI. *J Korean Neurosurg Soc* 46**,** 365-369.

Power, J.D., Barnes, K.A., Snyder, A.Z., Schlaggar, B.L., and Petersen, S.E. (2012). Spurious but systematic correlations in functional connectivity MRI networks arise from subject motion. *Neuroimage* 59**,** 2142-2154.

Power, J.D., Schlaggar, B.L., and Petersen, S.E. (2015). Recent progress and outstanding issues in motion correction in resting state fMRI. *Neuroimage* 105**,** 536-551.

Raven, J. (1958). *Standard progressive matrices.* London: Lewis & Co.

Smith, S.M., Jenkinson, M., Woolrich, M.W., Beckmann, C.F., Behrens, T.E., Johansen-Berg, H., Bannister, P.R., De Luca, M., Drobnjak, I., Flitney, D.E., Niazy, R.K., Saunders, J., Vickers, J., Zhang, Y., De Stefano, N., Brady, J.M., and Matthews, P.M. (2004). Advances in functional and structural MR image analysis and implementation as FSL. *Neuroimage* 23 Suppl 1**,** S208-219.

Strauss, E., Sherman, E.M.S., and Spreen, O. (2006). *A compendium of neuropsychological tests. Administration, norms, and commentary (third edition)* New York: Oxford University Press.

Tzourio-Mazoyer, N., Landeau, B., Papathanassiou, D., Crivello, F., Etard, O., Delcroix, N., Mazoyer, B., and Joliot, M. (2002). Automated anatomical labeling of activations in SPM using a macroscopic anatomical parcellation of the MNI MRI single-subject brain. *Neuroimage* 15**,** 273-289.

Von Aster, M., Neubauer, A., and Horn, R. (2006). *WIE. Wechsler Intelligenztest für Erwachsene. Deutschsprachige Bearbeitung und Adaptation des WAIS II von David Wechsler.* Frankfurt a. M.: Harcourt Test Services.

Wechsler, D. (1997). *Wechsler Adult Intelligence Scale - Third Edition.* San Antonio, TX: The Psychological Corporation.

Yuan, W., Wade, S.L., and Babcock, L. (2014). Structural connectivity abnormality in children with acute mild traumatic brain injury using graph theoretical analysis. *Hum Brain Mapp*.

Zalesky, A., Fornito, A., and Bullmore, E.T. (2010). Network-based statistic: identifying differences in brain networks. *Neuroimage* 53**,** 1197-1207.

Zimmermann, P., and Fimm, B. (2002). "A test battery for attentional performance," in *Applied Neuropsychology of Attention. Theory, Diagnosis and Rehabilitation,* eds. Leclercq M. & Z. P. (London: Psychology Press), 110-151.
